# BMPR2 Loss Activates AKT by Disrupting DLL4/NOTCH1 and PPARγ Signaling in Pulmonary Arterial Hypertension

**DOI:** 10.3390/ijms25105403

**Published:** 2024-05-15

**Authors:** Keytam S. Awad, Shuibang Wang, Edward J. Dougherty, Ali Keshavarz, Cumhur Y. Demirkale, Zu Xi Yu, Latonia Miller, Jason M. Elinoff, Robert L. Danner

**Affiliations:** 1 Critical Care Medicine Department, Clinical Center, NIH, Bethesda, MD 20892, USA; swang@cc.nih.gov (S.W.); edward.dougherty@fda.hhs.gov (E.J.D.); alikeshavarz235@gmail.com (A.K.); cumhur.demirkale@nih.gov (C.Y.D.); latoniakmiller@yahoo.com (L.M.); rdanner@nih.gov (R.L.D.); 2 Critical Care Medicine and Pulmonary Branch, National Heart, Lung, and Blood Institute, NIH, Bethesda, MD 20892, USA; yuz@nhlbi.nih.gov (Z.X.Y.); jason.elinoff@nih.gov (J.M.E.)

**Keywords:** DLL4, BMPR2, PPARγ, AKT, pulmonary arterial hypertension, endothelial dysfunction, vascular remodeling, signal transduction

## Abstract

Pulmonary arterial hypertension (PAH) is a progressive cardiopulmonary disease characterized by pathologic vascular remodeling of small pulmonary arteries. Endothelial dysfunction in advanced PAH is associated with proliferation, apoptosis resistance, and endothelial to mesenchymal transition (EndoMT) due to aberrant signaling. DLL4, a cell membrane associated NOTCH ligand, plays a pivotal role maintaining vascular integrity. Inhibition of DLL4 has been associated with the development of pulmonary hypertension, but the mechanism is incompletely understood. Here we report that *BMPR2* silencing in pulmonary artery endothelial cells (PAECs) activated AKT and suppressed the expression of DLL4. Consistent with these in vitro findings, increased AKT activation and reduced DLL4 expression was found in the small pulmonary arteries of patients with PAH. Increased NOTCH1 activation through exogenous DLL4 blocked AKT activation, decreased proliferation and reversed EndoMT. Exogenous and overexpression of DLL4 induced *BMPR2* and PPRE promoter activity, and *BMPR2* and *PPARG* mRNA in idiopathic PAH (IPAH) ECs. PPARγ, a nuclear receptor associated with EC homeostasis, suppressed by BMPR2 loss was induced and activated by DLL4/NOTCH1 signaling in both *BMPR2*-silenced and IPAH ECs, reversing aberrant phenotypic changes, in part through AKT inhibition. Directly blocking AKT or restoring DLL4/NOTCH1/PPARγ signaling may be beneficial in preventing or reversing the pathologic vascular remodeling of PAH.

## 1. Introduction

Pulmonary arterial hypertension (PAH) is a progressive cardiopulmonary disease notable for vascular remodeling with obstruction and rarefaction of small pulmonary arteries [1]. These functional and structural changes produce sustained increases in pulmonary vascular resistance, ultimately leading to right heart failure and death. Currently available therapies targeting the endothelin, nitric oxide and prostacyclin pathways have reduced symptoms and prolonged patient survival. However, stopping or reversing disease progression has remained elusive [2,3]. Early endothelial injury and loss [4] followed by endothelial dysfunction with AKT activation [5,6,7,8] and a proliferative, anti-apoptotic phenotype are central hallmarks of PAH vascular pathology [9].

The PI3K/AKT signaling pathway has important roles in endothelial cell survival and proliferation [10]. The PI3K family of lipid kinases consists of two subunits, the regulatory p85 subunit and the catalytic p110 subunit. Class I PI3Ks (α, β, and δ) control cell growth and apoptosis, through the second messenger phosphatidylinositol (3,4,5)-triphosphate (PIP3) [11]. PIP3 recruits AKT to the plasma membrane where it can be fully activated by PDK1 and mTORC2. In endothelial cells (ECs), the proliferative and anti-apoptotic activity of AKT is also regulated by the NOTCH pathway [12]. Four transmembrane NOTCH receptors (NOTCH1-4) interact with five membrane-bound ligands (DLL1, DLL3, DLL4, JAGGED1 and JAGGED2) resulting in two cleavage events culminating in the release of the NOTCH intracellular domain (NICD) [13]. NICD translocates to the nucleus where it heterodimerizes with the DNA binding protein RBP-J (recombination signal binding protein for immunoglobulin kappa J region) to regulate gene transcription. NOTCH signaling is an essential pathway that maintains vascular integrity by promoting endothelial cell cycle arrest [14], restricting endothelial inflammation [15] and stabilizing cell-to-cell junctions [16,17]. Mice with targeted disruption of *Dll4* or *Notch1* die early in gestation of vascular defects.

Arterial endothelium predominantly expresses DLL4 [18], a NOTCH1 and NOTCH4 ligand. DLL4 is repressed by TNFα [19] and IFNγ [20] inflammatory cytokines implicated in idiopathic PAH (IPAH) and disease-associated PAH (APAH) [21,22]. Notably, anti-DLL4 monoclonal antibodies, developed to treat cancer, have been associated with pulmonary hypertension (PH) in clinical trials [23,24,25,26]. Conversely, DLL4/NICD activation by olmesartan, an angiotensin II receptor blocker, attenuated transverse aortic constriction-induced cardiac remodeling in mice [27] and monocrotaline-induced pulmonary hypertension and right ventricular hypertrophy in rats [28]. Importantly, BMPR2 was required for NOTCH1 activation and deletion of endothelial specific *Notch1* in transgenic mice exacerbated hypoxia-induced pulmonary hypertension [29]. Recently, Wang et al. implicated NICD loss in the development of PAH, finding decreased NICD expression in mouse models of pulmonary hypertension and in PAH patients [30].

While DLL4/NOTCH1 signaling appears to protect the pulmonary vasculature, how and under what conditions DLL4 loss or inhibition contributes to the development of pulmonary hypertension and pathologic vascular remodeling is not understood. Since AKT activation is associated with the development of PAH [5,6,7] and DLL4/NOTCH1, like BMPR2 [31] and PPARγ [32], appear to be protective [33,34], the noncanonical crosstalk and interdependent regulation of these pathways were investigated in healthy and *BMPR2*-silenced PAECs, as well as in the lungs of patients with heritable PAH (HPAH) and IPAH. The interactions among these molecules were extensively bidirectional, such that perturbation of one affected the others, each contributing to aberrant signaling and endothelial phenotypic changes commonly associated with PAH pathogenesis.

## 2. Results

### 2.1. Silencing BMPR2 Activates AKT and Blocks Apoptosis

**Figure 1:**
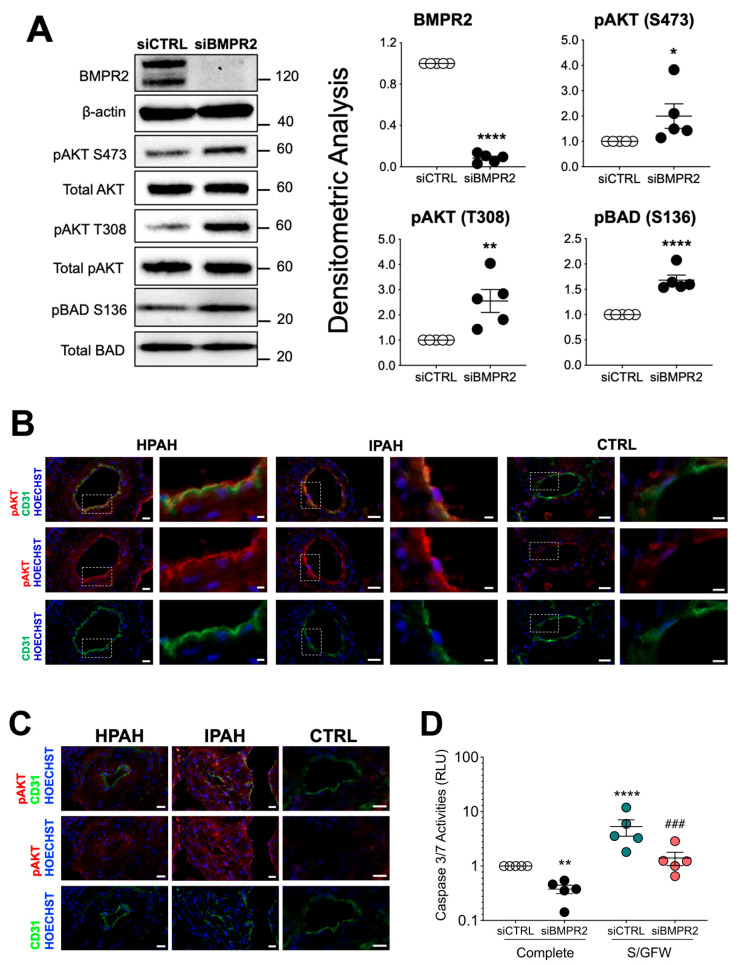
BMPR2 knockdown activates AKT and protects the cells against apoptosis. (**A**) Human primary pulmonary artery endothelial cells were transfected with control (siCTRL) or BMPR2 siRNA (siBMPR2) for 48 h and total protein lysates were collected. Western blots of BMPR2, phosphorylation of AKT at S473 and T308, total AKT, phosphorylation of BCL2-associated agonist of cell death (BAD) at S136 and total BAD were detected. Densitometric analysis of each protein relative to total β-actin, total AKT or total BAD and normalized to its corresponding siCTRL. Representative Western blots are shown, *n* = 5 independent experiments. (**B**,**C**) Immunofluorescence of pAKT (S473) (red), CD31 (green) antibodies and nuclear staining with Hoechst 33,342 (blue) in HPAH, IPAH and healthy control (CTRL) lung. Scale bar = 20 μm. Insert scale bar = 5 μm. (**D**) Caspase 3/7 activity was measured after 24 h withdrawal of serum and growth factors (S/GFW). Data presented as mean ± SEM; *n* = 5. (**A**) Paired *t*-test, * *p* < 0.05; ** *p* < 0.01; **** *p* < 0.001; (**D**) 2-way ANOVA with Tukey HSD **^###^** *p* < 0.005 (siBMPR2-complete vs. siBMPR2- S/GFW).

Heterozygous loss-of-function (LOF) mutations in *BMPR2* are the leading cause of HPAH and account for 10–40% of cases without a family history [35]. Aberrant activation of the PI3K/AKT pathway has been associated with endothelial and smooth muscle cell proliferation as well as apoptosis resistance in late-stage PAH [5,6,7]. Therefore, the effect of BMPR2 loss on phosphorylation of both AKT and BCL2-associated agonist of cell death (BAD) were examined in PAECs after 48 h of knockdown (Figure 1A). *BMPR2* silencing significantly activated AKT as shown by phosphorylation of both S473 and T308, while BAD, a target of AKT, was phosphorylated at S136 which inactivates its pro-apoptotic effects. Compared to control lung tissue, immunofluorescence staining of small pulmonary arteries in lung tissue from PAH patients showed increased phosphorylation of AKT (S473) co-localized with CD31, an endothelial cell marker (Figure 1B; for patient characteristics, see Supplementary Table S1). Activation of AKT was also increased in plexogenic lesions as compared to control (Figure 1C). Caspase 3/7 activity, a marker of apoptosis, was suppressed in *BMPR2*-silenced PAECs grown in complete media and also after the induction of apoptosis by serum and growth factor withdrawal (Figure 1D).

### 2.2. JNK1 Suppression Contributes to Apoptosis Resistance

**Figure 2:**
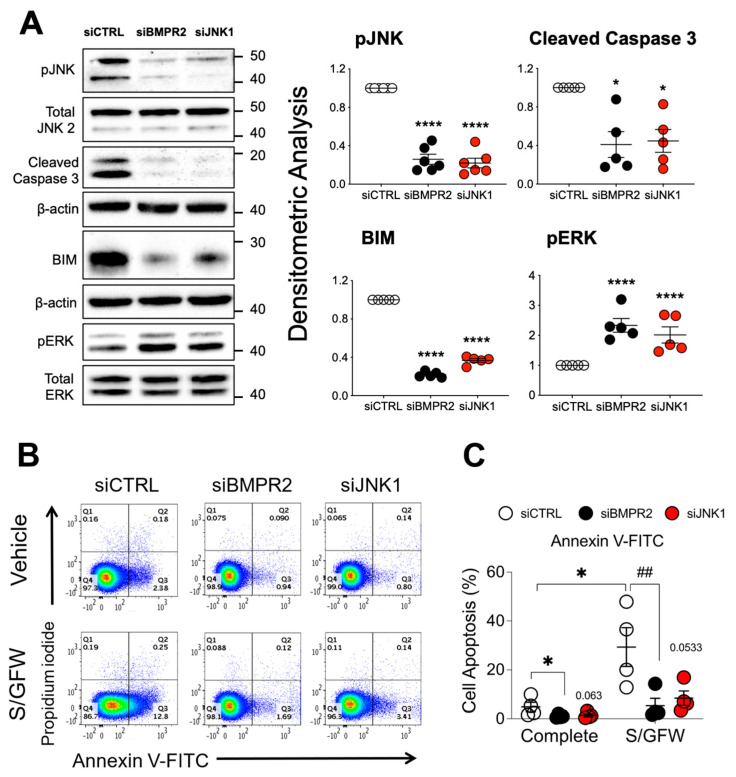
Loss of activated JNK1 contributes to apoptosis resistance as measured by reduced caspase 3 cleavage and flow cytometry analysis. (**A**) Human primary pulmonary artery endothelial cells (PAECs) were transfected with control (siCTRL), BMPR2 (siBMPR2), or JNK1 (siJNK1) siRNA for 48 h and total protein lysates were collected and analyzed for phosphorylated JNK1 (pJNK), cleaved caspase 3, BIM and phosphorylated ERK (pERK). Densitometric analysis was performed relative to total JNK2, β-actin or total ERK and normalized to its corresponding siCTRL. Representative Western blots are shown (*n* = 5, 6 independent experiments). (**B**) After siRNA transfection (48 h), cells were cultured in either complete media or serum and growth factor-free media (S/GFW) for 24 h followed by staining with annexin and propidium iodide (PI) for 10 min and data acquired on MACSquant. (**C**) Column scatter plot representing the percentage of apoptotic cells (cells stained with annexin V not PI, quadrant 3) (*n* = 4). Data presented as mean ± SEM; (**A**) 1-way ANOVA with Tukey HSD, * *p* < 0.05; **** *p* < 0.001; (**C**) 2-way ANOVA with Tukey HSD; ^##^ *p* < 0.01 (siCTRL- S/GFW vs. siBMPR2- S/GFW).

We previously observed that BMPR2 loss in PAECs decreased JNK1 phosphorylation [36] which is a known downstream consequence of AKT activation [37]. As JNK1 activation can trigger apoptosis, its suppression was examined as a possible contributor to the apoptosis-resistant endothelial phenotype. Silencing *BMPR2* or *JNK1* (Figure 2A) similarly reduced both cleaved caspase 3, a measure of caspase 3 activity, and proapoptotic BIM protein (Bcl-2 interacting mediator of cell death or BCL2L11), while increasing ERK phosphorylation/activation, a pro-survival signal. Moreover, flow cytometry analysis of annexin V and propidium iodide (AV/PI) staining of apoptotic cells showed that JNK1 knockdown, like BMPR2 loss, protected cells from serum and growth factor withdrawal-induced apoptosis (Figure 2B,C).

### 2.3. Leniolisib Attenuates AKT Activation, Inhibits Cell Proliferation and EndoMT, and Induces Apoptosis in BMPR2 and CAV1 Silenced PAECs

Because AKT activation and signaling have been implicated in PAH pathogenesis through its pro-survival, anti-apoptotic functions [5,6,7], PI3K/AKT was targeted to determine whether its blockade would ameliorate aberrant phenotypic changes associated with BMPR2 loss. Leniolisib, a well-tolerated PI3K inhibitor recently approved by the FDA for the treatment of children with activated phosphoinositide 3-kinase delta (PI3Kδ) syndrome (APDS) [38,39], was tested in PAECs after knockdown of either *BMPR2* or *CAV1*.

**Figure 3:**
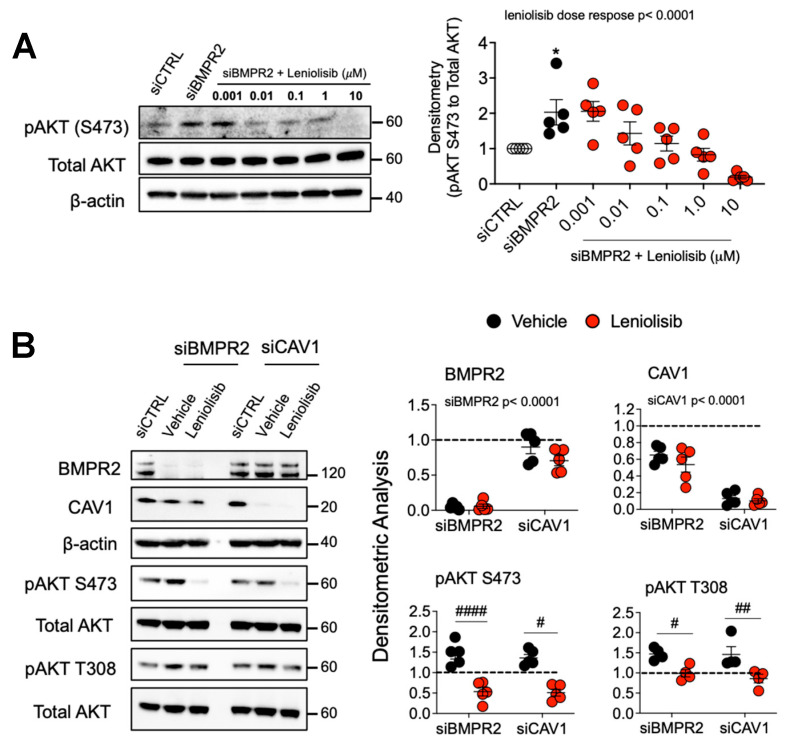

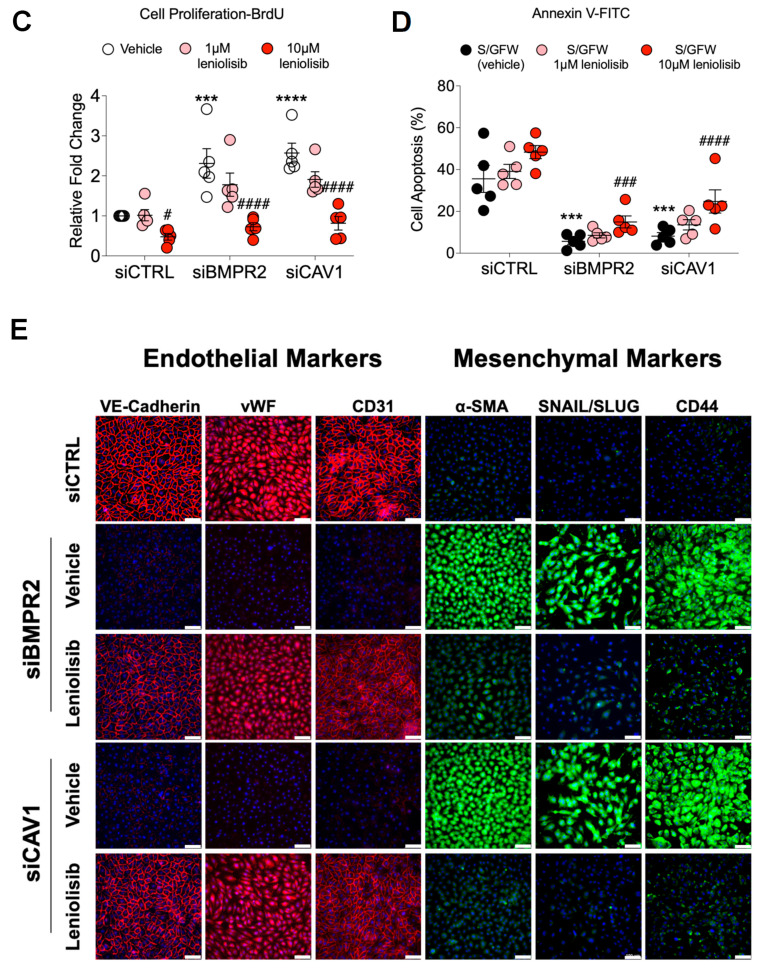
PI3Kδ inhibitor, leniolisib, decreases AKT activation, proliferation, and EndoMT while increasing apoptosis in *BMPR2*-silenced PAECs. (**A**) Human primary pulmonary artery endothelial cells (PAECs) were transfected with control (siCTRL) or BMPR2 (siBMPR2) siRNA for 48 h and then exposed to leniolisib for 4 h at the indicated concentrations (*n* = 5). (**B**) Effect of 10 μM leniolisib on phosphorylation of AKT (S473 and T308) in *BMPR2*- and *CAV1*-silenced PAECs. Representative Western blots are shown and densitometric analysis (mean ± SEM) relative to β-actin or total AKT and normalized to its corresponding siCTRL (*n* = 4, 5). (**C**) BrdU cell proliferation of PAECs transfected with control (siCTRL), BMPR2 (siBMPR2) or CAV1 (siCAV1) siRNA for 48 h then replated and treated with vehicle (DMSO), 1 μM or 10 μM leniolisib for an additional 72 h. (**D**) Leniolisib reactivated apoptosis as assessed by annexin and PI staining (*n* = 5). (**E**) Representative immunofluorescence of endothelial markers (VE-cadherin, vWF and CD31) and mesenchymal markers (α-SMA, SNAIL/SLUG and CD44) in PAECs transfected with control, BMPR2, or CAV1 siRNA for 48 h then treated with 10 μM leniolisib for 24 h (*n* = 5). Scale bar = 100 μm. Data presented as mean ± SEM. * *p* < 0.05 (siCTRL versus siBMPR2), ^#^ *p* < 0.05 (vehicle versus treatment).

In human PAECs, leniolisib dose-dependently decreased AKT activation in both our *BMPR2* and *CAV1* [40] LOF in vitro models of PAH (Figure 3A,B). Next, we determined the effect of leniolisib on cell proliferation, EndoMT and apoptosis, all of which are characteristic of endothelial dysfunction in PAH [41,42]. Cell proliferation in both the *BMPR2* and *CAV1* LOF models [36,40] was reduced by leniolisib at concentrations that were achievable in patients with activated PI3Kδ syndrome [38] (Figure 3C). Importantly, leniolisib dose-dependently increased apoptosis as measured by AV/PI staining (Figure 3D) and enhanced caspase 3/7 activity in both the *BMPR2* and *CAV1* in vitro LOF models of PAH (Supplementary Figure S1). Forty-eight hours after *BMPR2* or *CAV1* silencing, PAECs were treated with leniolisib for 24 h then stained for EndoMT markers. PAECs exhibited reduced expression of endothelial markers (VE-cadherin, vWF and CD31) and enhanced expression of mesenchymal markers (α-SMA, SNAIL/SLUG and CD44) as assessed by immunofluorescence. All of these changes were significantly reversed by leniolisib (Figure 3E; fluorescence intensity of each EndoMT marker was quantified and plotted in Supplementary Figure S2). Taken together, these results demonstrate that inhibiting AKT blocks proliferation, reverses EndoMT and induces apoptosis.

### 2.4. Loss of DLL4/NOTCH1 Signaling in BMPR2-Silenced PAECs and in PAH Lung and Its Reactivation Blocks AKT, Suppressing Cell Proliferation

**Figure 4:**
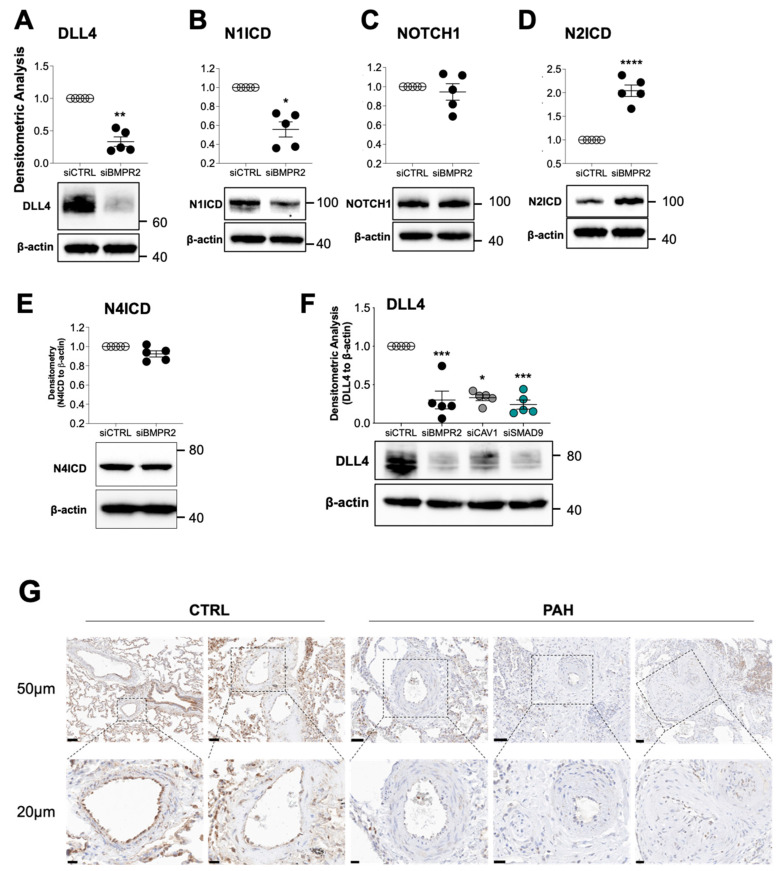
DLL4 is decreased in BMPR2-silenced PAECs and in lung tissue from patients with pulmonary arterial hypertension (PAH). Human primary pulmonary artery endothelial cells (PAECs) were transfected with control (siCTRL) or BMPR2 (siBMPR2) siRNA for 48 h and total protein lysates were collected for Western blotting of (**A**) DLL4, (**B**) N1ICD, (**C**) NOTCH1, (**D**) N2ICD, and (**E**) N4ICD. (**F**) DLL4 protein was also analyzed in PAECs transfected with either control (siCTRL) or BMPR2 (siBMPR2), CAV1 (siCAV1) or SMAD9 (siSMAD9) gene-specific siRNA pools. Representative Western blots are shown and densitometric analysis relative to β-actin and normalized to its corresponding siCTRL (*n* = 5). (**G**) Immunohistochemical staining of DLL4 in paraffin embedded lung of failed donor controls (CTRL; *n* = 5), HPAH (*n* = 3) and IPAH (*n* = 2). Scale bar = 50 μm. Insert scale bar = 20 μm Data are presented as mean ± SEM; *n* = 5. (**A**–**E**) paired *t*-test, (**F**) 1-way ANOVA with Tukey HSD * *p* < 0.05; *** *p* < 0.005; and **** *p* < 0.001.

Since AKT can also be regulated by NOTCH signaling and the loss of N1ICD has recently been implicated in PAH pathogenesis [30], we analyzed the expression of DLL4, a membrane-bound NOTCH ligand, and NOTCH receptors that are known to be expressed in endothelial cells. *BMPR2* silencing in PAECs reduced DLL4 protein (Figure 4A) and N1ICD (Figure 4B), the transcriptionally active, intracellular effector of NOTCH1 signaling. Nonetheless, total NOTCH1 was unchanged (Figure 4C), while N2ICD was increased (Figure 4D). N4ICD was also unchanged in *BMPR2*-silenced PAECs (Figure 4E). Note that N3ICD was not examined because NOTCH3 is not expressed in endothelium.

Like *BMPR2*-silenced PAECs, DLL4 expression was similarly reduced in PAECs following *CAV1* or *SMAD9* siRNA knockdown (Figure 4F). Notably, compared to healthy lung vessels, DLL4 protein expression was reduced in the small pulmonary artery endothelium of PAH lungs (Figure 4G; for patient characteristics see Supplementary Table S2). DLL4 protein was also decreased in neointima and adventitia layers of occluded vessels. For an overview of DLL4 staining in lung of five healthy controls, three HPAH patients and two IPAH patients, see Supplementary Figure S3.

**Figure 5:**
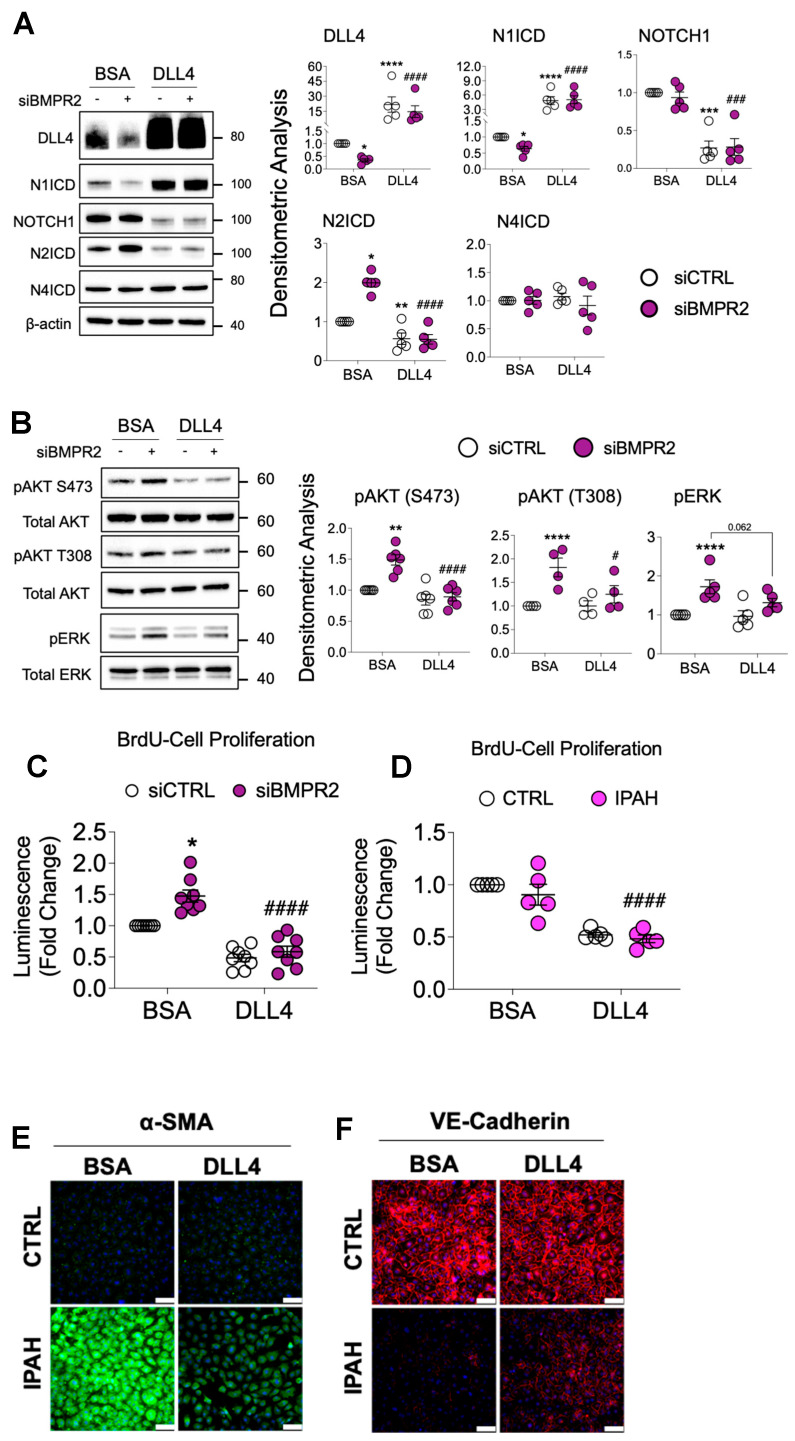
Immobilized DLL4 activates NOTCH1 signaling inhibiting AKT activation, proliferation and EndoMT. Human primary pulmonary artery endothelial cells (PAECs) were grown on BSA- or DLL4-coated plates and the following day the cells were transfected with either non-targeting control (siCTRL) or BMPR2 (siBMPR2) gene-specific siRNA pools. After BMPR2 knockdown (48 h), total protein lysates were collected and analyzed by Western blotting for expression of (**A**) DLL4, N1ICD, NOTCH1, N2ICD and N4ICD or (**B**) phosphorylated AKT (pAKT S473 and pAKT T308) or phosphorylated ERK (pERK). Representative Western blots are shown (*n* = 4–6, as indicated). Densitometric analysis relative to β-actin, total AKT or total ERK and normalized to its corresponding siCTRL. (**C**) BrdU cell proliferation of PAECs transfected with control or BMPR2 siRNA for 48 h and then replated onto BSA- or DLL4-coated plates for an additional 48 h (*n* = 5). (**D**) BrdU cell proliferation of healthy and IPAH ECs were grown on either BSA or DLL4 for 48 h (*n* = 5). (**E**,**F**) Immunofluorescence staining of α-SMA (ACTA2) and VE-Cadherin (CDH5) in ECs from healthy and IPAH grown on either BSA or DLL4. Scale bar = 100 μm. Data are presented as mean ± SEM, 2-way ANOVA with Tukey HSD; * *p* < 0.05; ** *p* < 0.01; **** *p* < 0.001 (siCTRL-BSA versus siBMPR2-BSA); **^#^** *p* < 0.05; **^###^** *p* < 0.005; and **^####^** *p* < 0.001 (siBMPR2-BSA versus siBMPR2-DLL4).

DLL1, also a membrane-bound NOTCH ligand like DLL4, has been reported to mimic cell-to-cell interactions and activate NOTCH signaling [43] when immobilized on plastic cell culture plates. Therefore, the same approach was adopted to investigate DLL4 in cultured PAECs. Compared to BSA, immobilized DLL4 markedly increased DLL4 protein in total cell lysates, activated NOTCH1 signaling as indicated by the accumulation of N1ICD and decreased NOTCH1 in both the absence and presence of *BMPR2* silencing (Figure 5A).

**Figure 6:**
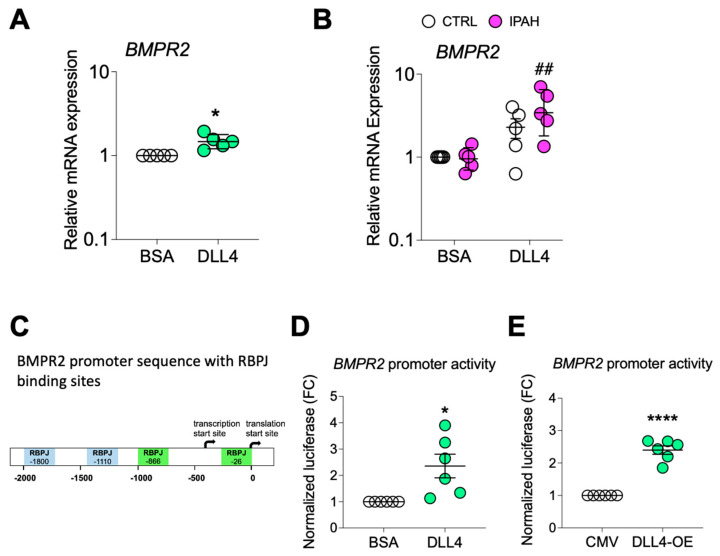
DLL4 increases *BMPR2* transcription. (**A**,**B**) Quantitative RT-PCR of *BMPR2* mRNA from (**A**) PAECs or from (**B**) healthy or IPAH PAECs grown on BSA- or DLL4-coated plates. (**C**) Schematic of *BMPR2* promoter with putative RBPJ (recombination signal binding protein for immunoglobulin kappa J region) binding sites indicated. (**D**) BMPR2 promoter activity in PAECs grown on BSA- or DLL4-coated plates and transfected with the promoter driven luciferase reporter. (**E**) BMPR2 promoter activity in PAECs transfected with the promoter driven luciferase reporter plus either empty vector control (CMV) or DLL4 overexpression plasmid (DLL4-OE). For quantitative RT-PCR, data are presented as the geometric mean ± SD; *n* = 4, 5. For promoter activity, data presented as mean ± SEM; *n* = 6. Paired *t*-test, * *p* < 0.05; **** *p* < 0.001; and **^##^** *p* < 0.01 (siBMPR2-BSA versus siBMPR2-DLL4).

Although immobilized DLL4 also blocked increases in N2ICD (Figure 5A), this is unlikely to mediate the DLL4-driven EC phenotypes as NOTCH2/N2ICD suppression promotes proliferation and inhibits apoptosis in ECs [44,45]. N4ICD was not affected, indicating that DLL4-mediated NOTCH signaling was occurring primarily through NOTCH1 and not NOTCH4. Activation of NOTCH1 signaling by immobilized DLL4 blocked both AKT and ERK activation (Figure 5B) and reduced cell proliferation in *BMPR2*-silenced cells (Figure 5C). Cell proliferation was also compared between failed donor control (CTRL) and IPAH PAECs. Although no difference was detected between these two groups when cultured ex vivo on BSA-coated plates, exogenous DLL4 significantly inhibited proliferation in both (Figure 5D). Immobilized DLL4 also significantly abrogated α-SMA (ACTA2) expression as assessed by immunofluorescence staining in PAECs from IPAH patients compared to failed donor controls (Figure 5E). Conversely, expression of VE-cadherin (CDH5) in IPAH PAECs was modestly enhanced by DLL4 (Figure 5F). Fluorescence intensity of α-SMA and VE-cadherin was quantified and plotted in Supplementary Figure S4A. *BMPR2* silencing and IPAH PAECs grown on immobilized DLL4 also demonstrated reduced α-SMA protein as compared to BSA (Supplementary Figure S4B,C). In addition to reversal of EndoMT marker expression, growth on DLL4-coated plates also resulted in increased *BMPR2* mRNA expression in PAECs (Figure 6A) regardless of whether these cells were from failed donor controls or IPAH patients (Figure 6B). Notably, TRANSFAC^®^ analysis of the *BMPR2* promoter identified multiple binding motifs for the Notch pathway transcription factor (recombination signal binding protein for immunoglobulin kappa J region; RBPJ) (Figure 6C), suggesting that NOTCH signaling may directly regulate *BMPR2* transcription. In support of this, immobilized DLL4 exposure (Figure 6D) and *DLL4* overexpression (Figure 6E) each activated a reporter gene driven by the *BMPR2* promoter indicating that DLL4/NOTCH1 signaling may maintain endothelial homeostasis in part by increasing BMPR2. DLL4 overexpression and N1ICD activation was verified in PAECs (Supplementary Figure S5).

### 2.5. DLL4/N1ICD Upregulation of PPARγ Expression and Signaling Reduces AKT Activation

**Figure 7:**
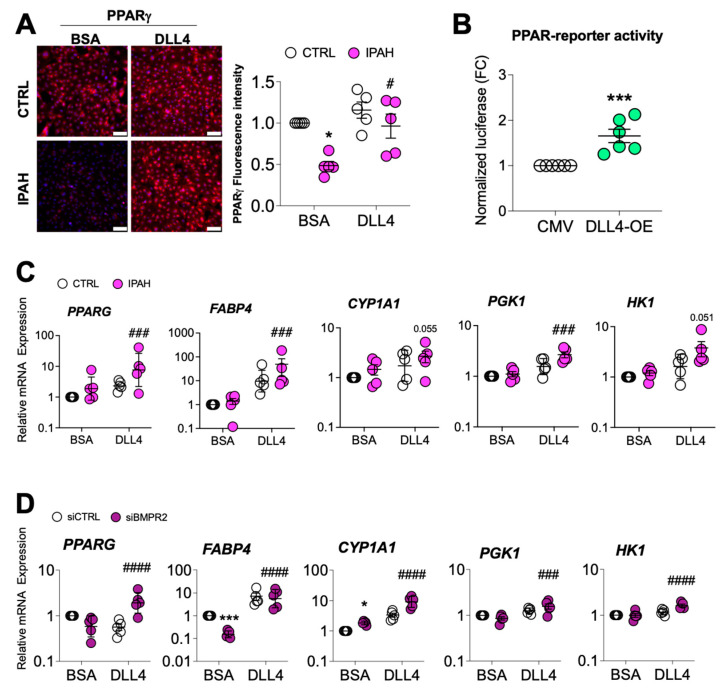

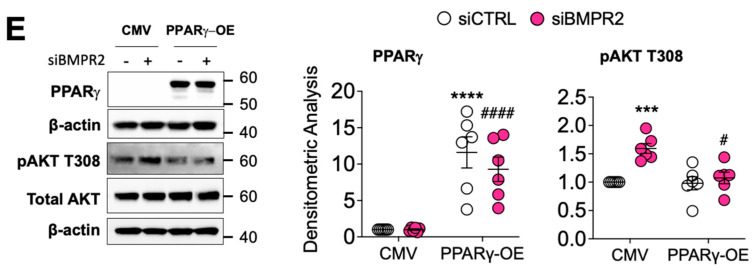
DLL4-induced NOTCH1 activation rescues PPARγ expression. (**A**) PPARγ immunofluorescence staining of PAECs endothelial cells from healthy or IPAH patients grown on either BSA or DLL4 (*n* = 5). Scale bar = 100 μm. (**B**) Human primary pulmonary artery endothelial cells (PAECs) were transfected with empty vector (CMV) or DLL4 overexpression plasmid (DLL4-OE) for 24 h and PPAR-driven reporter activity assessed (*n* = 6). (**C**) mRNA levels from healthy (CTRL) or IPAH ECs grown on BSA or DLL4 for 48 h were analyzed for *PPARG* expression and PPARγ target genes. (**D**) mRNA from PAECs transfected with control (siCTRL) or BMPR2 (siBMPR2) siRNA and grown on BSA or DLL4 for 48 h were analyzed for *PPARG* expression and PPARγ target genes. (**E**) After 24 h of gene silencing with either control or BMPR2 siRNAs, PAECs were then transfected with empty vector control (CMV) or PPARγ overexpression plasmid for 24 h and expression of PPARγ and phosphorylated AKT (pAKT T308) protein was analyzed by Western blotting. Densitometric quantification relative to β-actin or total AKT and normalized to its corresponding siCTRL. Representative Western blots are shown. Data presented as mean ± SEM; *n* = 6. The mRNA levels were measured by quantitative RT-PCR and presented as the geometric mean ± SD; *n* = 5. 2-way ANOVA with Tukey HSD; * *p* < 0.05; *** *p* < 0.005 (siCTRL-BSA versus siBMPR2-BSA); **^#^** *p* < 0.05; **^###^** *p* < 0.005; and **^####^** *p* < 0.001 (siBMPR2-BSA versus siBMPR2-DLL4).

Loss of PPARγ in ECs leads to a proliferative, apoptosis-resistant phenotype [34] similar to the phenotype seen following the loss of BMPR2. PPARγ is also a downstream effector of BMPR2 [33], therefore we next examined whether DLL4-mediated NOTCH1 signaling affected PPARγ expression. Notably, immunofluorescence staining for PPARγ protein in PAECs from IPAH patients was decreased, while DLL4-induced N1ICD signaling restored PPARγ expression in these cells (Figure 7A). Furthermore, PAECs transfected with a *DLL4* expression vector, and a PPAR-driven reporter showed that DLL4/N1ICD signaling increased the transcription of PPARγ target genes (Figure 7B). As expected, overexpression of *DLL4* increased *DLL4* mRNA levels similar to exposure to immobilized DLL4, and increased *PPARG* mRNA (Supplementary Figure S6A,B). Accordingly, in PAECs from IPAH patients (Figure 7C) and in *BMPR2*-silenced PAECs (Figure 7D), DLL4 not only induced *PPARG* transcription, but also increased mRNA expression of several PPARγ target genes (*FABP4*, *CYP1A1*, *PGK1* and *HK1*). Since immobilized DLL4 not only blocks AKT activation but also induces PPARγ genomic signaling, we directly examined the impact of PPARγ overexpression on AKT phosphorylation in human PAECs. Overexpression of PPARγ was found to decrease AKT phosphorylation at T308 (Figure 7E), implicating PPARγ as a mechanistic link between DLL4-mediated NOTCH1 signaling and AKT inhibition.

## 3. Discussion and Conclusions

*BMPR2* LOF mutations are the most frequent genetic defect associated with the development of PAH [46,47] and reduced BMPR2 expression is also common in IPAH [48]. Endothelial dysfunction manifested by proliferation, apoptosis resistance and EndoMT contributes to small pulmonary artery remodeling, a hallmark of late-stage PAH progression [41,42]. Here, BMPR2 loss disrupted DLL4/NOTCH1 signaling, activating AKT and suppressing PPARγ expression, a protective gatekeeper that mitigates against the development PAH. While BMPR2 deficiency reduced DLL4 protein and DLL4/NOTCH1 signaling, two other in vitro models of PAH based on *CAV1* or *SMAD8/9* LOF were also associated with reduced DLL4 expression. More importantly, small pulmonary arteries from patients with HPAH and IPAH both exhibited elevated AKT activation and reduced DLL4 expression compared to vessels from donor lungs. Accordingly, exogenous or overexpressed DLL4 both suppressed AKT activation, while inducing itself, *BMPR2*, and *PPARG* in PAECs, including those from IPAH patients. Likewise, the overexpression of PPARγ, presumably downstream from DLL4 was also found to suppress AKT activation in *BMPR2*-silenced PAECs. Finally, exogenous DLL4 or leniolisib, a PI3Kδ/AKT inhibitor, were shown to prevent the aberrant PAH-like endothelial phenotype associated with BMPR2 loss. These novel findings demonstrate extensive crosstalk between the DLL4/NOTCH1 and BMPR2/PPARγ networks in the regulation of AKT, supporting the development of strategies to activate DLL4/NOTCH1/PPARγ and/or inhibit AKT signaling for the treatment of pathologic vascular remodeling in PAH.

Vascular remodeling of small pulmonary arteries is attributed to hyper-proliferation, EndoMT and apoptosis-resistance in part caused by dysregulated PI3K/AKT signaling [6]. We found increased AKT activation in BMPR2-silenced PAECs and in the pulmonary arteries of patients with either HPAH or IPAH lung. A recent study demonstrated that inhibition of the PI3Kα isoform was found to reverse PH in a mouse model of chronic hypoxia, the sugen/hypoxia and MCT-induced rat model by effectively decreasing growth-factor-induced phosphorylation of AKT in vascular smooth muscle cells [49]. However, PI3Kα signaling pathway has been demonstrated to have a cardioprotective role as chronic inhibition of PI3Kα accelerated the progression of heart failure [50]. Given that inflammation and excessive PI3K/AKT signaling are common features of various PAH models [40,51] and inflammatory cytokines like TNFα can specifically induce the expression of PI3Kδ in ECs, we targeted the PI3Kδ isoform. Leniolisib is a well-tolerated, orally available PI3Kδ inhibitor, recently approved by the FDA for the treatment of children with activated PI3Kδ syndrome caused by a gain-of-function mutation in the PI3Kδ gene (PIK3CD) [38]. We found that leniolisib effectively blocked AKT activation in *BMPR2*-silenced PAECs and partially normalized cell proliferation, EndoMT and apoptosis resistance, and phenotypic features associated with end stage vascular remodeling in PAH.

DLL4 monoclonal antibodies were recently reported to cause pulmonary vascular remodeling and PH in mice through the inhibition of endothelial NOTCH1 signaling [30]. LOF or haploinsufficiency of *DLL4* [52] or *NOTCH1* [53] lead to Adams–Oliver syndrome, which includes PAH as one of its manifestations. Interestingly, *SOX17* deficiency, a risk factor for the development of IPAH, HPAH and PAH associated with congenital heart disease [54], was reported to transcriptionally induce DLL4 and protect against vascular leakage [55]. In fact, loss of endothelial specific *SOX17* [56] in mice mimicked *DLL4* haploinsuffiency [57] suggesting that *SOX17* mutations might cause PAH in part by disrupting DLL4 signaling. However, this hypothesis has not been tested. In the present study, we found reduced DLL4 and N1ICD protein expression in *BMPR2*-silenced PAECs. Importantly, DLL4 loss was not specific to BMPR2 deficiency and was also seen in our *CAV1* and *SMAD8*/9 LOF models of PAH, and in lung tissue from patients with HPAH and IPAH. This commonality indicates that disrupted DLL4/NOTCH1 signaling might be a broadly applicable paradigm in PAH pathogenesis. Furthermore, we demonstrate that DLL4, as expected, activated NOTCH1/N1ICD signaling but also suppressed AKT activation, while reducing cell proliferation and EndoMT in *BMPR2*-silenced PAECs or IPAH ECs. This normalization of endothelial function occurred even in the presence of PAH-associated genetic deficiencies. In support of this, DLL4 or Jagged 1 activation of NOTCH1 was found to inhibit proliferation by repressing the PI3K/AKT and ERK signaling pathways in human iliac artery endothelial cells [12]. Other reports not supporting this conclusion warrant mention. For example, in human PAECs and umbilical endothelial cells, γ-secretase inhibitors, which block the proteolytic cleavage and release of intracellular N1ICD from the NOTCH1 receptor, were demonstrated to decrease cell proliferation and survival, right ventricular systolic pressure, and right heart hypertrophy in sugen/hypoxia rats [58,59]. However, these discrepant results might be due to the nonselectivity of γ-secretase inhibitors, targeting other ligand/NOTCH pairs and not just DLL4/NOTCH1. Additionally, γ-secretase may regulate other receptors, such that γ-secretase inhibitors might have non-NOTCH, off-target effects. Notably, DLL4 levels were not measured, and exogenous DLL4 was not used in these investigations.

How anti-DLL4 monoclonal antibodies caused PH in patients undergoing cancer treatment is not known. In addition to the likelihood, based on our results that DLL4/NOTCH1 blockade would likely de-repress AKT activation and reduce PPARγ, we hypothesized that DLL4/N1ICD signaling might regulate BMPR2 expression. Since N1ICD does not directly bind to DNA, but instead interacts with the transcription factor RBPJ to regulate the expression of NOTCH target genes [60], we explored the BMPR2 promoter for potential RBPJ binding motifs. Notably, two putative RBPJ binding motifs were identified in the *BMPR2* promoter. Exogenous DLL4 increased *BMPR2* mRNA in PAECs from both failed donor controls and IPAH patients. In addition, the overexpression of DLL4 in PAECs activated a reporter gene driven by the *BMPR2* proximal promoter. These findings suggest that BMPR2 deficiency, the most common heritable defect associated with PAH, could be exacerbated or caused by loss or inhibition of DLL4/NOTCH1 signaling.

In human macrophages, DLL4/NOTCH1/N1ICD was found to increase the stability of PPARγ [61], a known downstream effector of BMPR2 [33] that has anti-inflammatory and anti-proliferative functions in the vasculature [62] and has been described to guard against the development of PAH [32]. Reduced PPARγ expression was demonstrated in the lung tissue of patients with PAH and implicated in the proliferative, anti-apoptotic behavior of PAECs from these patients [34]. Mice with targeted endothelial [63] or smooth muscle cell [33] deletion of *Pparγ* spontaneously developed PAH with increased right ventricular hypertrophy and muscularization of the distal pulmonary arteries, whereas restoration of PPARγ has been reported to reverse experimental PH [63,64]. Furthermore, in the lung tissue of monocrotaline-treated rats, PPARγ blocked AKT phosphorylation at S473 through the upregulation of PTEN [65], a dual protein/lipid phosphatase that mainly targets PI3K/AKT pathway. In this study, DLL4/N1ICD restored PPARγ levels in ECs isolated from patients with IPAH and also increased *PPARG* mRNA and PPARγ target gene expression in *BMPR2*-silenced PAECs as well as in ECs from IPAH patients. Importantly, PPARγ expression induced by DLL4/N1ICD signaling inhibited AKT activation associated with BMPR2 loss. These results indicate that the disruption of DLL4/NOTCH1 signaling could exacerbate the development of PAH through AKT activation along with reduced BMPR2 and PPARγ expression. The underling mechanisms by which PPARγ inhibits AKT activation and ways to augment this important function of PPARγ warrant further investigation.

**Figure 8:**
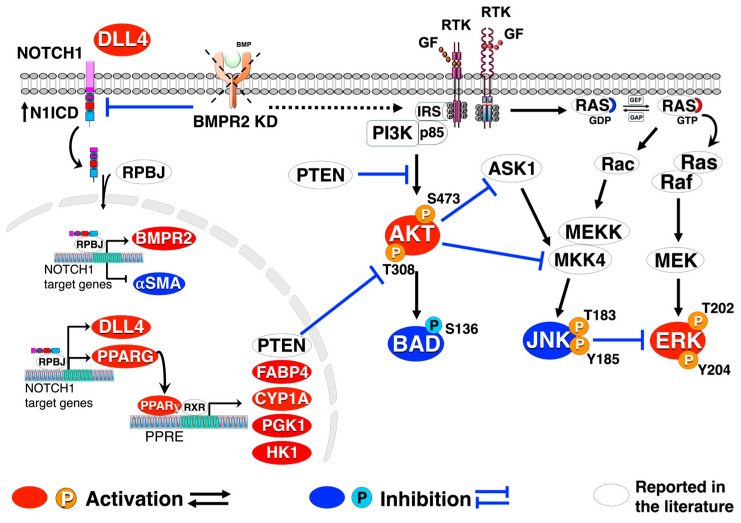
Reactivation of DLL4/NOTCH1 signaling blocks AKT activation, inhibiting proliferation and endothelial-to-mesenchymal transition. Crosstalk among DLL4/NOTCH1, BMPR2, PPARɣ, and AKT signaling pathways. BMPR2 knockdown (KD) culminates in sustained AKT and ERK activation along with JNK1 inactivation leading to an apoptosis-resistant, hyper-proliferative, endothelial-to-mesenchymal transition (EndoMT). Loss of BMPR2 also results in decreased DLL4/N1ICD and PPARɣ protein. Activation of DLL4/N1ICD signaling increases BMPR2/PPARɣ expression blocking AKT activation leading to a decrease in proliferation and EndoMT.

In conclusion, we found that BMPR2 deficiency disrupts DLL4/NOTCH1 signaling, activates AKT, ERK and inhibits JNK. Furthermore, PPARγ is identified as an important downstream effector of the DLL4/NOTCH1 signaling (Figure 8). Loss of DLL4/NOTCH1 and PPARγ signaling in BMPR2-silenced ECs contribute to the activation of AKT and recapitulates many features of PAH pathobiology. Restoring DLL4/NOTCH1/PPARγ signaling and/or suppressing AKT activation may be beneficial in preventing or reversing the pathologic vascular remodeling of PAH.

## 4. Materials and Methods

### 4.1. Cell Culturing

Primary PAECs (Lonza, Frederick, MD, USA) were cultured for a maximum of 6 passages on cell culture flasks coated with type I collagen (50 μg/mL in 0.02 N acetic acid; Corning, Corning, NY, USA) in endothelial basal medium-2 (EBM™-2, Lonza, Frederick, MD, USA) supplemented with growth factors and 2% serum (EGM™-2 SingleQuot kit, Lonza, Frederick, MD, USA). Failed donor control (CTRL) and IPAH endothelial cells (ECs) obtained from the Pulmonary Hypertension Breakthrough Initiative (PHBI) (see Supplementary Table S3 donor information) were plated on 0.1% gelatin (Sigma, St. Louis, MO, USA). In experiments studying exogenous DLL4 effects, cells were seeded on plates pre-coated with recombinant DLL4 (0.5 μg/mL in PBS overnight, R&D Systems; Minneapolis, MN, USA). Cells were maintained at 37 °C in a humidified incubator with 5% CO_2_ and 95% air.

### 4.2. Gene Silencing

Primary PAECs were transfected with gene-specific siRNA pools targeting *BMPR2, JNK1*, *CAV1 or SMAD9* at a final concentration of 10 nM using DharmaFECT-1 (Dharmacon; Lafayette, CO, USA) in Opti-MEM (Invitrogen; Grand Island, NY, USA) for 6 h followed by growth in EGM™-2 for 48 h. Non-targeting siRNA Pool-1 (siGENOME, Dharmacon, Lafayette, CO, USA) was used as a control.

### 4.3. Immunofluorescence

Explanted lung tissue from unused donor controls, IPAH and HPAH subjects were obtained from PHBI (see Supplementary Table S1 for donor information). Frozen lung sections were air dried, fixed with fresh 4% paraformaldehyde for 10 min, washed 3× with PBS at room temperature (RT) for 10 min and permeabilized with 0.02% Trition X-100 for 5 min. Tissue sections were briefly dipped in PBS, blocked with 10% donkey serum for 1 h in the dark with gentle shaking and incubated with anti-CD31 and anti-pAKT (S473) overnight at 4 °C. Sections were gently washed 3× with PBS, incubated with Alexa fluor 488 donkey anti-mouse and Alexa fluor 647 donkey anti-rabbit for 1 h at RT, washed 3x with PBS and stained with Hoechst 33,342 for 15 min. Tissue sections were mounted with Prolong Diamond Antifade Mountant (ThermoFisher Scientific, Rockford, IL, USA) and scanned using the Zeiss Axioscan Z1 (Zeiss, White Plains, NY, USA).

Primary PAECs were seeded onto collagen-coated 8-well chamber slides (Sigma) at a density of 60,000 cells/well overnight, transfected with *BMPR2*, *CAV1* or control siRNA for 48 h and treated with vehicle (DMSO) or leniolisib (Novartis, Basel, Switzerland) for 24 h. Failed donor control or IPAH ECs (50,000 cells/well) were seeded on either BSA- or DLL4-coated 8-well chamber slides for 48 h. Cells were then washed 3× with basal medium, fixed with 4% formaldehyde (Polysciences, Inc., Warrington, PA, USA) for 20 min at RT, blocked with goat serum (Sigma, St. Louis, MO, USA) plus 0.3% Triton X-100 for 45 min at RT and then washed 3× with PBS supplemented with 0.1% BSA before incubating overnight with primary antibodies. The next day, the slides were washed 3× as above, incubated with secondary antibodies for 1 h, washed 3× as above and mounted using mounting medium with DAPI (4′,6-diamidino-2-phenylindole; Sigma, St. Louis, MO, USA). Cells were imaged using epifluorescence (Leica Dmi8 Inverted LED Fluorescence Microscope, Leica, Deerfield, IL, USA) at 20× magnification and analyzed using ImageJ V 1.8.0.

### 4.4. Immunohistochemistry

Explanted human lung tissue from unused donor controls, IPAH and HPAH subjects were obtained from PHBI (see Supplementary Table S2 for donor information). Paraffin embedded lung tissue sections (10 μm) were cut (Histoserv, Inc., Germantown, MD, USA) and slides were stained for expression of DLL4 at the Lombardi Comprehensive Cancer Center Histopathology and Tissue Shared Resource (Georgetown University Medical Center, Washington, DC, USA). Briefly, tissue sections were deparaffinized with xylene, rehydrated through a graded alcohol series and epitope retrieval performed in a Dako PT Link (Agilent, Santa Clara, CA, USA) using a 1x LowFlex Target Retrieval solution from DAKO #K8005. For immunohistochemical staining, tissue slides were treated with 3% hydrogen peroxide plus 10% normal goat serum for 10 min, incubated overnight with DLL4 antibody at 1:300 (Abcam, Waltham, MA, USA, catalog #ab176876), and exposed to horseradish peroxidase (HRP)-labeled polymer for 30 min and DAB chromagen for 5 min. Tissue slides were then counterstained with Hematoxylin blue (Fisher Chemical^TM^ Harris Modified Hematoxylin) in 1% ammonium hydroxide, dehydrated and mounted with Acrymount. Negative controls without primary antibody were used.

### 4.5. Overexpression Studies

Primary PAECs were seeded at 30,000 cells/well on collagen-coated 24-well plates for 48 h followed by 24 h transfection with expression vectors for DLL4 (DLL4/pCMV6-myc-DDK) or empty plasmid (pCMV6-myc-DDK, Origene, Rockville, MD, USA) along with a *BMPR2* promoter or PPAR-luciferase reporter and pGL4.74 [hRluc/TK, *Renilla*-luciferase] plasmid (Promega, Madison, WI, USA) using Lipofectamine^TM^ Stem reagent (Invitrogen; Grand Island, NY, USA). The *BMPR2* promoter was designed by cloning the −1200/+100 human *BMPR2* promoter into the pGL4.15[luc2p/Hygro] vector (Promega, Madison, WI, USA). The PPAR reporter, containing 3 copies of PPAR response element upstream of the reporter gene 3-thymidine kinase-luciferase (PPAR-LUC), was kindly provided by Dr. Ronald M. Evans [66]. PAECs were also seeded on either BSA- or DLL4-coated plates for 48 h then transfected with the *BMPR2* promoter-luciferase reporter for an additional 24 h. Lysates were harvested using 1x passive lysis buffer (Promega, Madison, WI, USA) and analyzed using the Dual-Luciferase reporter assay system (Promega, Madison, WI, USA). Data was normalized to Renilla-luciferase from the same sample.

For protein lysates, PAECs were plated on either BSA or DLL4 overnight followed by *BMPR2* silencing for 24 h. The cells were then transfected with either empty plasmid (CMV) or PPARγ (PPARγ/pCMV6-myc-DDK) for 24 h before adding RIPA buffer (Invitrogen; Grand Island, NY, USA) supplemented with protease and phosphatase inhibitor cocktail (Invitrogen; Grand Island, NY, USA). Additionally, protein lysates were also collected 24 h after over-expressing empty plasmid or DLL4.

### 4.6. RNA Isolation, cDNA Synthesis and Quantitative Real-Time PCR

Total RNA was extracted using RNeasy Mini Kit (Qiagen, Valencia, CA, USA) according to the manufacturer’s instructions, including DNase I treatment. The iScript cDNA Synthesis Kit (Bio-Rad; Hercules, CA, USA) was used to synthesize cDNA and quantitative real-time PCR (qRT-PCR) analysis was performed using iTaq Universal SYBR Green Supermix with ROX (Bio-Rad) on a Biosystems ViiA^TM^7 instrument. Gene expression was normalized to β-actin and delta cycle thresholds were tested for significance. Primer sequences of target genes are listed in Supplementary Table S4.

### 4.7. Western Blot

Whole cell protein lysates from PAECs, unused failed donor and IPAH cells were lysed with RIPA buffer supplemented with protease and phosphatase inhibitor cocktail. Protein lysates (30 μg) fractionated by 4–12% SDS-PAGE were transferred on nitrocellulose membranes, blocked at RT for 1 h and incubated overnight at 4 °C with primary antibody (Supplementary Table S5). Blots were imaged with ChemiDoc MP Imaging System (BioRad, Hercules, CA, USA) and densitometry analysis performed with Image Lab software (version 6.1; BioRad).

### 4.8. Cell Proliferation and Cell Apoptosis Assays

Primary PAECs were transfected with siCTRL or siBMPR2 for 48 h in T75 flasks, detached with 0.5% trypsin-EDTA and re-seeded (5000 cells/well) on BSA- or DLL4-coated 96-well plates. Likewise, failed donor control or IPAH ECs were seeded on BSA- or DLL4-coated 96-well plates and cell proliferation over 72 h was quantitated by incorporation of 5-bromo-2-deoxyuridine (BrdU) using a chemiluminescence-based ELISA kit (Sigma, St. Louis, MO, USA). For apoptosis assays, primary PAECs transfected with either BMPR2 or control siRNA for 48 h were replated on collagen-coated 96-well plates at a density of 5000 cell/well overnight. The following day, media was replaced with either complete media or media without serum or growth factors for 24 h. An equal volume of Caspase-Glo 3/7 reagent (Promega) was added to each well for 30 min and luminescence was read with a GloMax^®^ plate reader (Promega). For flow cytometry, primary PAECs were plated on collagen-coated T25 flasks at a density of 220,000 cells/flask. The next day, cells were transfected with either control, *BMPR2*, *JNK1* or *CAV1* siRNA for 48 h. Cell media was then replaced with either complete media or media without serum or growth factors and the addition of either vehicle (DMSO) or leniolisib for another 24 h. Cells were harvested, stained with annexin V and propidium iodide (PI) (BD Pharmingen™) and evaluated using either a MACSquant^®^ (Miltenyi) or BD LSRFortessa™ (BD Biosciences) analyzer. Data files were then uploaded and analyzed using FlowJo software version 10.4.2 (Treestar, Ashland, OR, USA).

### 4.9. Statistics

Differences of relevant gene expression, protein expression/phosphorylation, caspase activity, apoptotic cells, cell migration, and BrdU incorporation between control and *BMPR2* siRNA transfected PAECs were analyzed by paired *t*-test. Differences between failed donor control and IPAH ECs were examined by unpaired *t*-test. ANOVA followed by post hoc Tukey’s honest significance test was used to analyze effects of *BMPR2*, *JNK1* or *CAV1* silencing and/or the efficacy of pathway inhibitors in PAEC apoptosis, proliferation, migration, gene expression and protein phosphorylation assays. Dose–responses across multiple conditions and donors were subjected to linear mixed models with random subject effects to account for the correlation within each donor. Log-transformation was applied when necessary. Two tailed *p* < 0.05 was accepted as significant. All statistical analyses were performed using JMP® software version 16.1.0 (SAS Institute Inc., Cary, NC, USA). Detailed sample sizes are described in the figure legends.

## Data Availability

The data that support these findings are available from the corresponding author upon reasonable request.

## Supplementary Materials

The following supporting information can be downloaded at: https://www.mdpi.com/article/10.3390/ijms25105403/s1.

## Abbreviations

BMPR2	Bone morphogenetic protein receptor 2
PAH	Pulmonary arterial hypertension
PAEC	Pulmonary arterial endothelial cells
PI3K	Phosphoinositide 3-kinase
NOTCH1	Neurogenic locus notch homolog protein 1
DLL4	Delta like canonical Notch ligand 4
BSA	Bovine serum albumin
PPARɣ	Peroxisome proliferator activated receptor gamma
BrdU	Bromodeoxyuridine
S/GFW	Serum: growth factor withdrawal
LOF	Loss of function
ERK	Extracellular signal-regulated kinases
EndoMT	Endothelial to mesenchymal transition
CAV1	Caveolin 1
SMAD9	Mothers against decapentaplegic homolog 9
RTK	Receptor tyrosine kinases
