# Supplementary material for: BMPR2 Loss Activates AKT by Disrupting DLL4/NOTCH1 and PPARγ Signaling in Pulmonary Arterial Hypertension
Source: Int J Mol Sci. 2024 May 15;25(10):5403. doi: 10.3390/ijms25105403 (PMC11121464; doi:10.3390/ijms25105403)
Supplement: Supplementary file 1 [file ijms-25-05403-s001.zip › Supplementary Figures and Tables.pdf]

BMPR2 Loss Activates AKT by Disrupting DLL4/NOTCH1 and PPARɣ Signaling in Pulmonary Arterial Hypertension

Keytam S. Awad, Shuibang Wang, Edward J. Dougherty, Ali Keshavarz, Cumhur Y. Demirkale,

Zu Xi Yu, Latonia Miller, Jason M. Elinoff, and Robert L. Danner

Supplementary Information

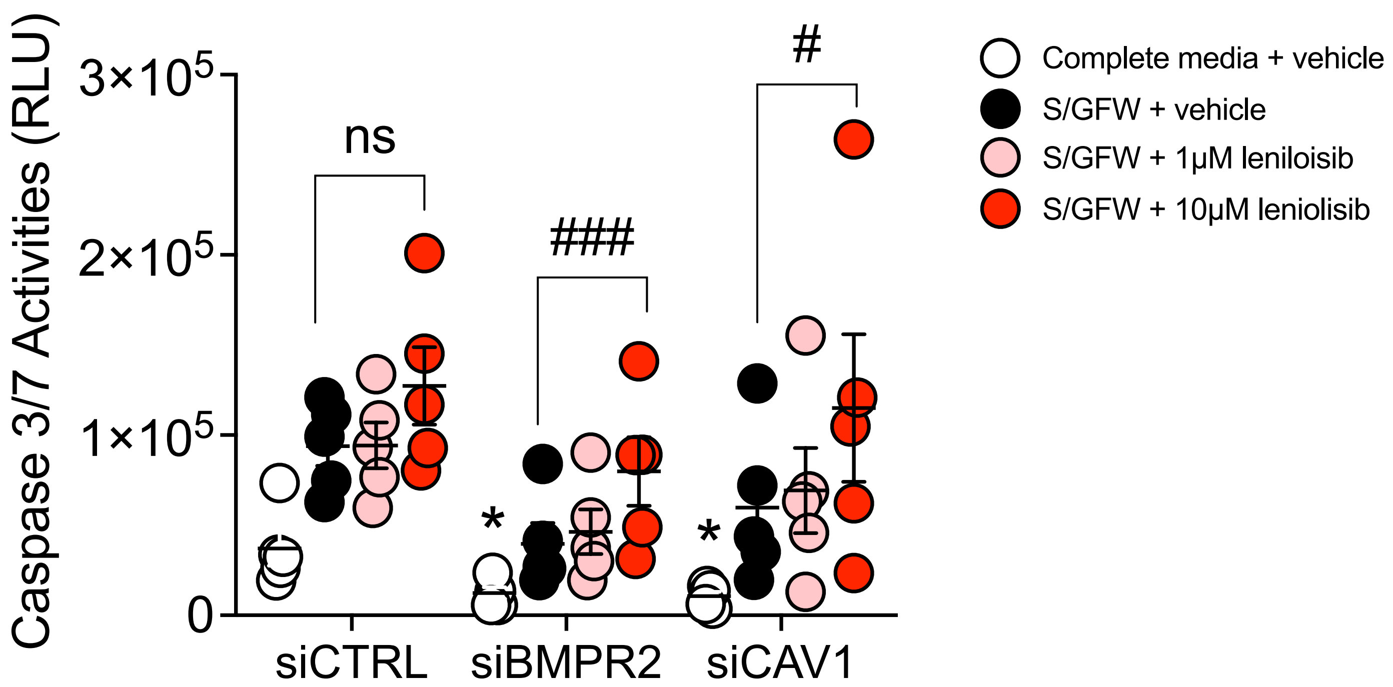

Figure S1. Leniolisib induces caspase 3/7 activity, a marker of apoptosis, in BMPR2- and CAV1-silenced human primary pulmonary artery endothelial cells (PAECs). PAECs were transfected with control (siCTRL), BMPR2 (siBMPR2) or CAV1 (siCAV1) siRNA for 48 h then replated and treated with vehicle (DMSO), serum growth factor free media (S/GFW) plus vehicle, S/GFW plus 1 μM leniolisib or S/GFW plus 10 μM leniolisib for an additional 24 h. Data are presented as mean ± SEM; n=5, 2-way ANOVA with Tukey HSD: *P < 0.05 (siCTRL-complete media + vehicle versus siBMPR2-complete media + vehicle or siCTRL-complete media + vehicle versus siCAV1-complete media + vehicle); ####P < 0.005 (siBMPR2-S/GFW + vehicle versus siBMPR2-S/GFW + 10 μM leniolisib); #P < 0.05 (siCAV1-S/GFW + vehicle versus siCAV1-S/GFW + 10 μM leniolisib).

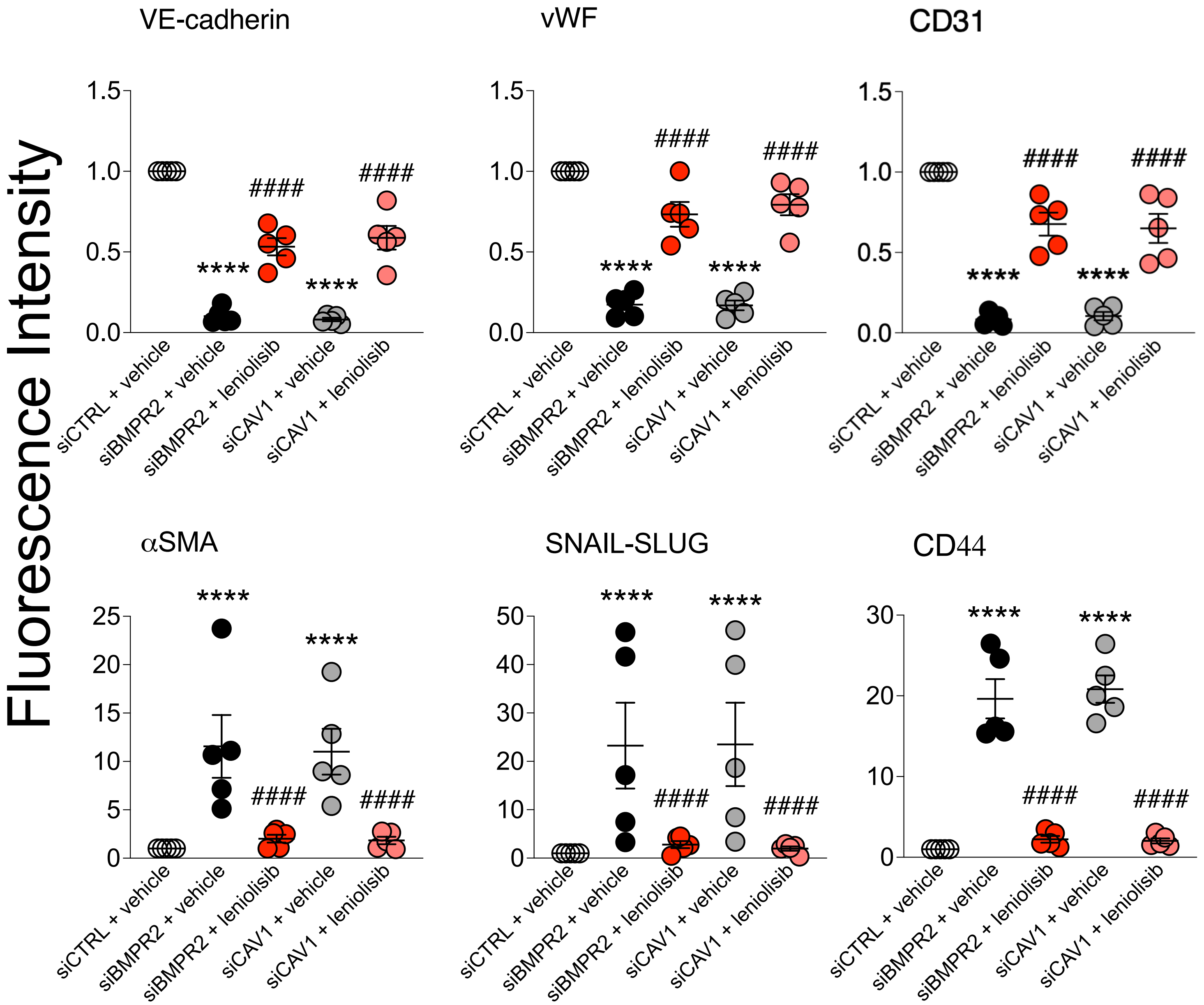

Figure S2. Leniolisib inhibits endothelial-mesenchymal transition (EndoMT) in *BMPR2* and *CAV1* silenced human primary pulmonary artery endothelial cells (PAECs). Fluorescence intensity of endothelial markers (VE-cadherin, vWF and CD31) and mesenchymal markers (α-SMA, SNAIL/SLUG and CD44) in PAECs transfected with control (siCTRL), BMPR2 (siBMPR2), or CAV1 (siCAV1) siRNA for 48 h. BMPR2- or CAV1-silenced cells were then incubated for an additional 24 h with or without 10 μM leniolisib. Data are presented as mean ± SEM; n = 5, 2-way ANOVA with Tukey HSD: ****$P < 0.001$ (siCTRL-vehicle versus siBMPR2-vehicle or siCTRL-vehicle versus siCAV1-vehicle); ####$P < 0.001$ (siBMPR2-vehicle versus siBMPR2-leniolisib or siCAV1-vehicle versus siCAV1-leniolisib).

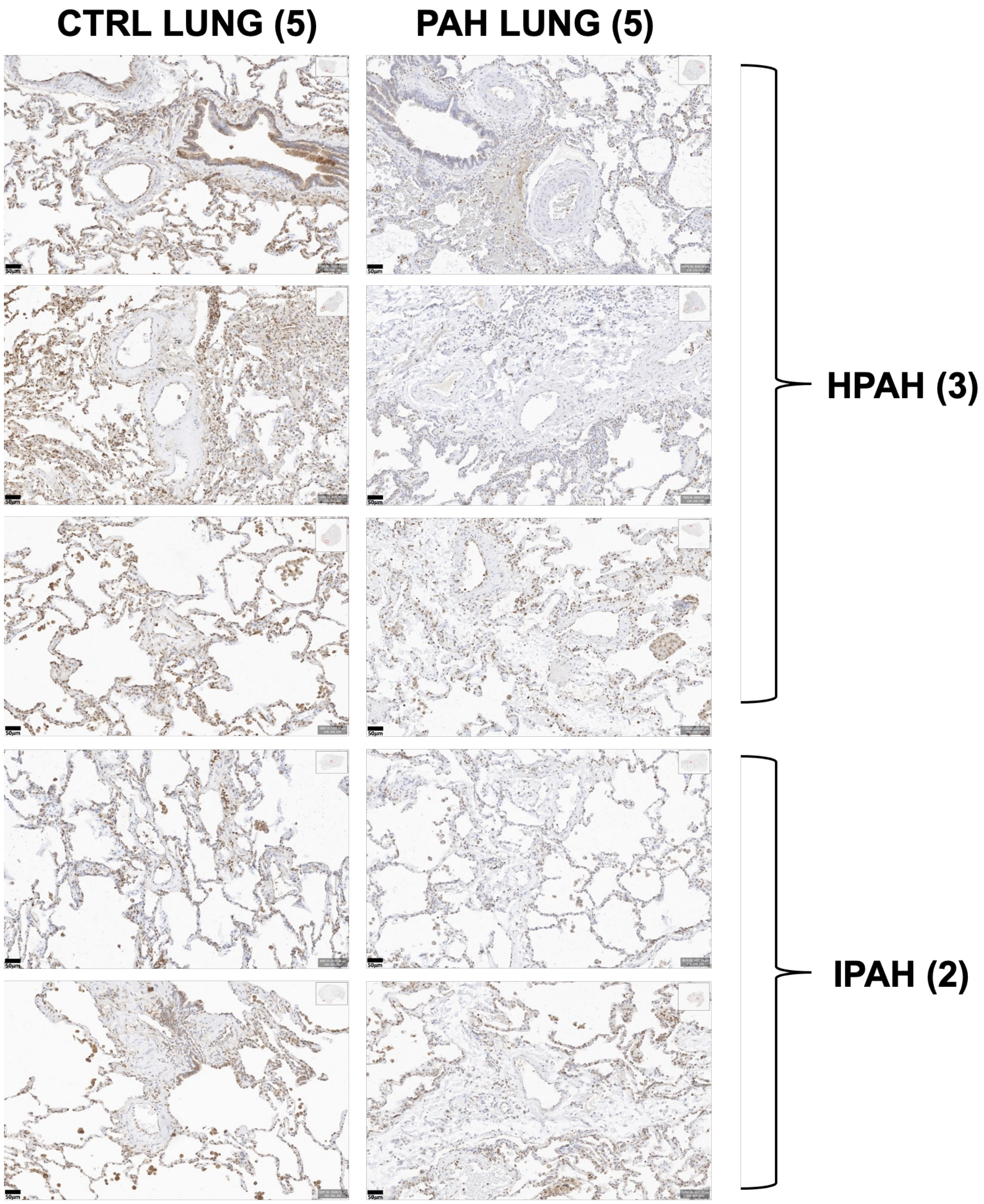

Figure S3. **DLL4 protein is reduced in lung of patients with PAH.** Immunohistochemical staining of DLL4 in paraffin embedded lung of failed donor controls (CTRL; n = 5), HPAH (n = 3) and IPAH (n = 2). Scale bar = 50µm.

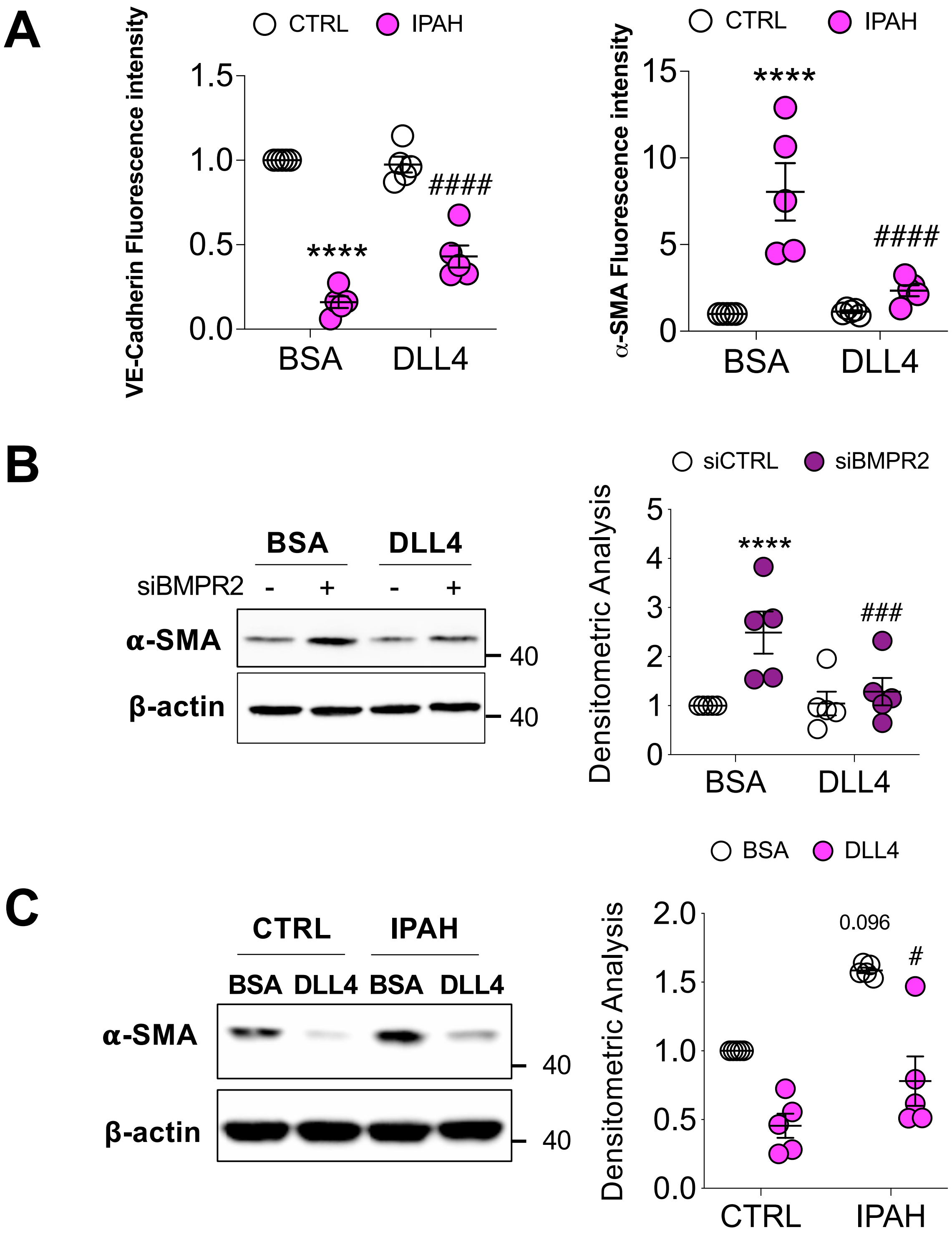

Figure S4. Exogenous DLL4 blocks α-SMA (ACTA2) induction in *BMPR2*-silenced human primary pulmonary artery endothelial cells (PAECs) and in PAECs from patients with idiopathic pulmonary arterial hypertension (IPAH). **(A)** Fluorescence intensity of VE-Cadherin (CDH5) and α-SMA (ACTA2) in IPAH ECs were grown on BSA or DLL4 coated plates for 48 h. **(B)** PAECs were grown on BSA or DLL4 coated plates and transfected with control (siCTRL) or BMPR2 (siBMPR2) siRNA for 48 h. **(C)** PAECs from failed donors (CTRL) and IPAH patients were grown on either BSA or DLL4 coated plates for 48 h. For both **B** and **C** total protein lysates were collected, and representative Western blots of α-SMA and β-actin are shown. Densitometric analyses of each protein relative to β-actin were normalized to its corresponding control. Data presented as mean ± SEM; n = 5, 2-way ANOVA with Tukey HSD: (A) ****$P < 0.001$ (CTRL-BSA versus IPAH-BSA); ####$P < 0.001$ (CTRL-DLL4 versus IPAH-DLL4); (B) ****$P < 0.001$ (siCTRL-BSA versus siBMPR2-BSA); ###$P < 0.005$ (siBMPR2-BSA versus siBMPR2-DLL4); (C) $P = 0.09$ (CTRL-BSA versus IPAH-BSA); #$P < 0.05$ (IPAH-BSA versus IPAH-DLL4).

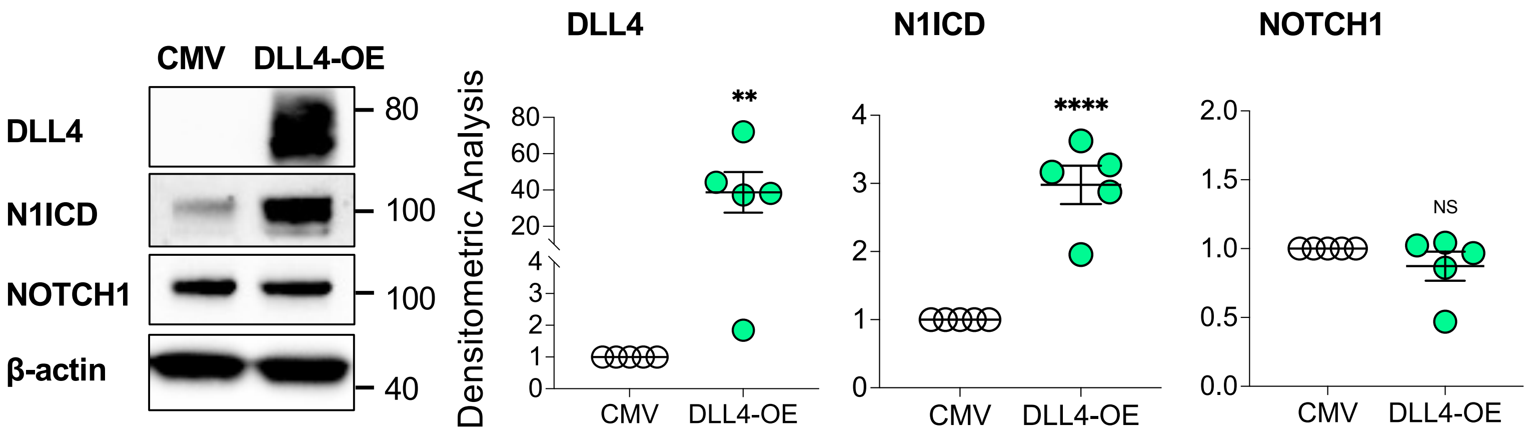

Figure S5. DLL4 overexpression increases N1ICD expression in primary human pulmonary artery endothelial cells (PAECs). PAECs were transfected with empty vector control (CMV) or DLL4 overexpression plasmid (DLL4-OE) for 24 h and total protein lysates were collected. Representative Western blots are shown for DLL4, N1ICD and NOTCH1. Densitometric analysis of each protein is relative to β-actin and normalized to its corresponding control. Data presented as mean ± SEM; n = 5, paired t-test: ***P* < 0.01; ****P < 0.001; NS, not significant.

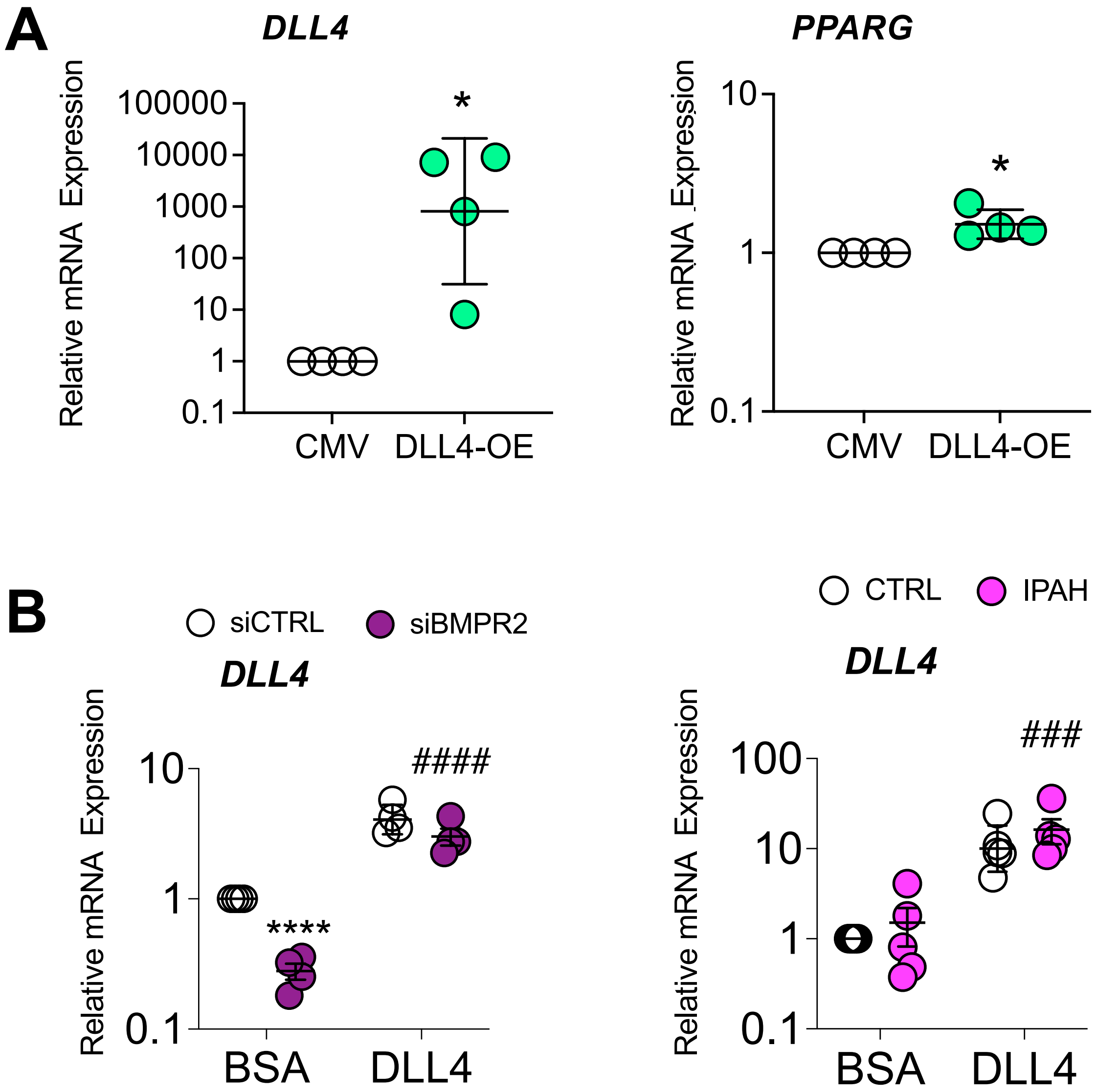

Figure S6. DLL4 overexpression or immobilized DLL4 increases DLL4 and PPARG mRNA in human primary pulmonary artery endothelial cells (PAECs) or cells from patients with idiopathic pulmonary arterial hypertension (IPAH). (A) Quantitative RT-PCR of DLL4 and PPARG mRNA in human primary pulmonary artery endothelial cells (PAECs) transfected with either empty vector control (CMV) or DLL4 overexpression plasmid (DLL4-OE) for 24 h, n = 4. **(B)** Quantitative RT-PCR of DLL4 in PAECs transfected with control (siCTRL) or BMPR2 (siBMPR2) siRNA, or PAECs from failed donors (CTRL) and IPAH patients grown on BSA or DLL4 coated plates, n = 4, 5. Data are presented as the geometric mean ± SD. (A) Paired t-test; *$P < 0.05$ (B) 2-way ANOVA with Tukey HSD: ****$P < 0.001$ (siCTRL-BSA versus siBMPR2-BSA); ####$P < 0.001$ (siBMPR2-BSA versus siBMPR2-DLL4); ###$P < 0.005$ (IPAH-BSA versus IPAH-DLL4).

Table S1: Patients' characteristics; Frozen Tissue

PHBI #	Age	Race	Gender	Diagnosis	BMPR2 Mutation
AH-022	57	White	Female	Failed Donor	No
AH-028	43	White	Female	Failed Donor	No
BA-037	60	White	Female	Failed Donor	No
BA-046	55	White	Female	Failed Donor	No
BA-054	33	Hispanic	Female	Failed Donor	No
BA-059	34	American Asian	Female	Failed Donor	No
UA-018	50	White	Female	Failed Donor	No
UA-020	51	Hispanic	Female	Failed Donor	No
CC-030	63	White	Female	IPAH	No
ST-001	10	Asian/Pacific Islander	Female	IPAH	No
ST-037	39	White	Female	IPAH	No
VA-014	33	White	Female	IPAH	Yes
CC-015	32	White	Female	HPAH	Yes
CC-032	54	White	Female	HPAH	Yes
UA-023	23	White	Female	HPAH	Yes
VA-010	33	Black	Female	HPAH	Yes

Table S2: Patients' characteristics for Immunohistochemistry

PHBI #	Age	Race	Gender	Diagnosis	BMPR2 Mutation
UA-018	50	White	Female	Failed Donor	No
UA-021	49	White	Female	Failed Donor	No
AH-022	57	White	Female	Failed Donor	No
BA-046	55	White	Female	Failed Donor	No
BA-059	34	American Asian	Female	Failed Donor	No
ST-012	26	American Asian	Female	IPAH	Yes
VA-014	33	White	Female	IPAH	Yes
VA-010	33	Black	Female	HPAH	Yes
UA-023	23	White	Female	HPAH	Yes
CC-033	25	White	Female	HPAH	Yes

Table S3: PHBI Subject Information

PHBI #	Age	Race	Gender	Diagnosis	Materials
UA-015	36	White	Female	Failed Donor	LMVEC
BA-037	60	White	Female	Failed Donor	LMVEC
BA-046	55	White	Female	Failed Donor	LMVEC
AH-022	57	White	Female	Failed Donor	LMVEC
BA-061	46	White	Female	Failed Donor	LMVEC
UA-018	50	White	Female	Failed Donor	LMVEC
CC-013	27	White	Female	IPAH	LMVEC
VA-011	32	White	Female	IPAH	LMVEC
ST-040	16	White	Female	IPAH	LMVEC
VA-017	40	White	Female	IPAH	LMVEC
ST-028	40	White	Female	IPAH	LMVEC
UA-026	55	White	Female	IPAH	LMVEC

Table S4: Quantitative real-time PCR primer sequences

Gene	Forward	Reverse
BMPR2	5′-CACTGCGGCTGCTTCGCAGA-3′	5′-AGCAGGTGCTACCTTTCGAGCA-3′
PPARγ	5′-GGATTCAGCTGGTCGATATCAC-3′	5′-GTTTCAGAAATGCCTTGCAGT-3′
FABP4	5′-ATCACATCCCCATTCACACT-3′	5′-ACTTGTCTCCAGTGAAAACTTTG-3′
CYP1A1	5′-TGGAGATTGGGAAAAGCATGA-3′	5′-GAACCTTCCCTGATCCTTGTG-3′
PGK1	5′-GACAGCAGCCTTAATCCTCTG-3′	5′-CTAACAAGCTGACGCTGGA-3′
HK1	5′-TCCCAACAATGAGTCCAACC-3′	5′-GCCACGATGTAGTCACCTTAC-3′
β-ACTIN	5′-CCGCCGCCAGCTCACCAT -3′	5′-ACCCATGCCCACCATCAGGC-3′
DLL4	MIQE CONTEXT SEQUENCE:<br>TGAGCAAACCAGCACCCTCACAAGG<br>CTGCGCTACTCTTACCGGGTCATCT<br>GCAGTGACAACTACTATGGAGACAA<br>CTGCTCCCGCCTGTGCAAGAAGCG<br>CAATGACCACTTCGGCCAC	
