# Supplementary material for: BMPR2 Loss Activates AKT by Disrupting DLL4/NOTCH1 and PPARγ Signaling in Pulmonary Arterial Hypertension
Source: Int J Mol Sci. 2024 May 15;25(10):5403. doi: 10.3390/ijms25105403 (PMC11121464; doi:10.3390/ijms25105403)
Supplement: Supplementary file 1 [file ijms-25-05403-s001.zip › Table S5. Antibody and Reagent List.pdf]

Table S5

REAGENT or RESOURCE	SOURCE	IDENTIFIER
**Antibodies and Reagents**		
BMPR2	BD Biosciences	612292, RRID:AB_399609
pAKT S473	Cell Signaling	9271S, RRID:AB_329825
pAKT T308	Cell Signaling	9275S, RRID:AB_329828
Total AKT	Cell Signaling	9272S, RRID:AB_329827
pERK	Cell Signaling	9101S, RRID:AB_331646
Total ERK	Cell Signaling	9102S, RRID:AB_330744
pJNK	Cell Signaling	4671S, RRID:AB_331338
Total JNK	Cell Signaling	9258S, RRID:AB_2141027
Cleaved CASPASE 3	Cell Signaling	9661S, RRID:AB_2341188
BCL2L11 (BIM)	Abcam	Ab7888, RRID:AB_2065161
NOTCH1, cleaved (Val1744) (N1ICD)	Cell Signaling	4147S, RRID:AB_2153348
NOTCH1	Cell Signaling	3608S, RRID:AB_2153354
NOTCH2	Cell Signaling	5732S, RRID:AB_10693319
N4ICD	Cell Signaling	2423S, RRID:AB_2151366
DLL4	Cell Signaling	96406S, RRID:AB_2800263
pBAD S136	Cell Signaling	4366S, RRID:AB_10547878
Total BAD	Cell Signaling	9292S, RRID:AB_331419
PPARγ	Cell Signaling	2443S, RRID:AB_823598
β-Actin (ACTB)	Sigma	A-3854, RRID:AB_262011
α-Smooth Muscle Actin (ACTA2)	Sigma	A-5228, RRID:AB_262054
CD31 (immunofluorescence)	BD BioSciences	555445, RRID:AB_395838
CD31 (immunofluorescence)	Agilent	M0823, RRID:AB_2114471
CDH5 (VE-cadherin; immunofluorescence)	Cell Signaling	2518S, RRID:AB_2077970
VWF (immunofluorescence)	Abcam	ab154193
VEGFR2 (immunofluorescence)	Cell Signaling	2479S, RRID:AB_10698606
CD44 (immunofluorescence)	Abcam	ab189524, RRID:AB_2885107
SNAI1/SLUG (immunofluorescence)	Abcam	ab167609
Horseradish peroxidase-conjugated goat anti-rabbit	Bio-Rad	170-6515, RRID:AB_11125142
Horseradish peroxidase-conjugated goat anti-mouse	Bio-Rad	170-6516, RRID:AB_11125547
Donkey anti-Mouse IgG (H+L) Cross-Adsorbed secondary, Alexa Fluor 488	ThermoFisher Scientific	A-21202, RRID:AB_141607
Donkey anti-Rabbit IgG (H+L) Cross-Adsorbed secondary, Alexa Fluor 647	ThermoFisher Scientific	A-32795, RRID:AB_2762835

Prolong Diamond Antifade Mountant	ThermoFisher Scientific	P36961
**Recombinant proteins**		
Recombinant Human DLL4 protein	R&D Systems	1506-D4-050
**Experimental models**		
Human pulmonary artery endothelial cells (HPAEC)	Lonza	CC-2530
IPAH, see Table S1	PHBI Penn Cell Center	02544
Non-Diseased (Controls), see Table S1	PHBI Penn Cell Center	02544
**Cell Culture**		
EGM-2 BulletKit (CC-3156 & CC-4176)	Lonza	CC-3162
EGM-2 MV BulletKit (CC-3156 & CC-4177)	Lonza	CC-3202
Opti-MEM	Gibco	31985077
**Critical commercial assays**		
Caspase-Glo 3/7	Promega	G8093
FITC Annexin V Apoptosis Detection Kit	BD Pharmingen	556547
Cell Proliferation ELISA, BrdU (chemiluminescent)	Sigma	11669915001
NE-PER Nuclear and Cytoplasmic Extraction kit	ThermoFisher Scientific	78835
Lipofectamine™ Stem reagent	ThermoFisher Scientific	STEM00001
iScript™ cDNA Synthesis Kit	Bio-Rad	170-8891
iTaq™ Universal SYBR® Green Supermix	Bio-Rad	1725124
**Oligonucleotides**		
Primers for qPCR, see Table S2		
**Recombinant DNA**		
Vector PPRE3-TK-LUC; 3 copies of PPRE upstream of the thymidine kinase promoter fused to the luciferase gene		Kindly provided by Ronald M. Evans
pGL4[luc2P/RBP-Jk-RE/Hygro]	Promega	CS173601
pGL4.15[luc2P/BMPR2/Hygro]	Promega	custom
pRL-TK Renilla luciferase	Promega	custom
PPARγ expression plasmid	Addgene	8895
DLL4 expression plasmid	Origene	custom
