# Supplementary material for: BMPR2 Loss Activates AKT by Disrupting DLL4/NOTCH1 and PPARγ Signaling in Pulmonary Arterial Hypertension
Source: Int J Mol Sci. 2024 May 15;25(10):5403. doi: 10.3390/ijms25105403 (PMC11121464; doi:10.3390/ijms25105403)

BMPR2 Loss Activates AKT by Disrupting DLL4/NOTCH1 and PPARɣ Signaling in Pulmonary Arterial Hypertension

Keytam S. Awad, Shuibang Wang, Edward J. Dougherty, Ali Keshavarz, Cumhur Y. Demirkale,

Zu Xi Yu, Latonia Miller, Jason M. Elinoff, and Robert L. Danner

Supplementary Information

Figure 1A

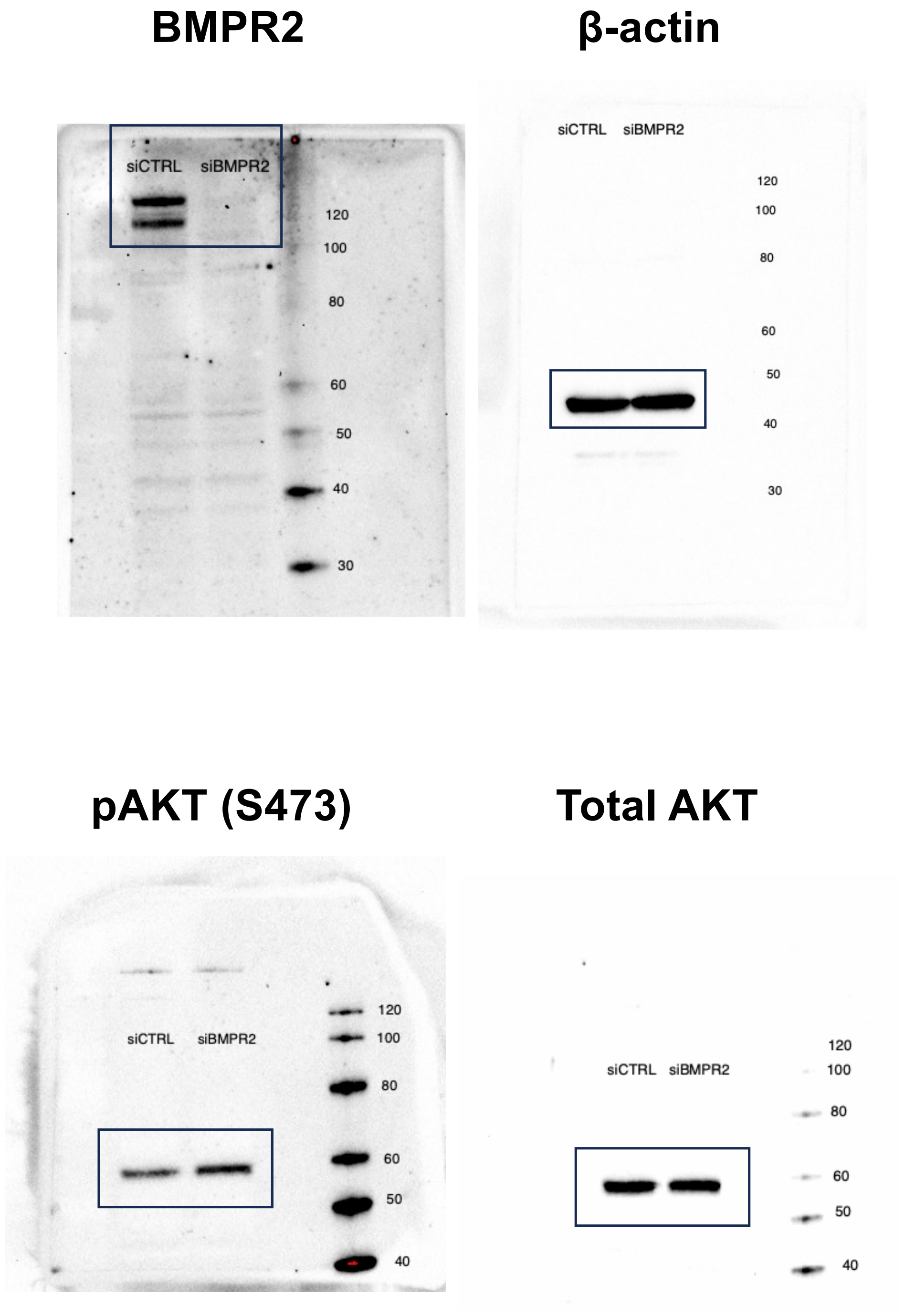

BMPR2
β-actin
siCTRL
siBMPR2
120
100
80
60
50
40
30
pAKT (S473)
Total AKT

Figure 1A

pAKT (T308)

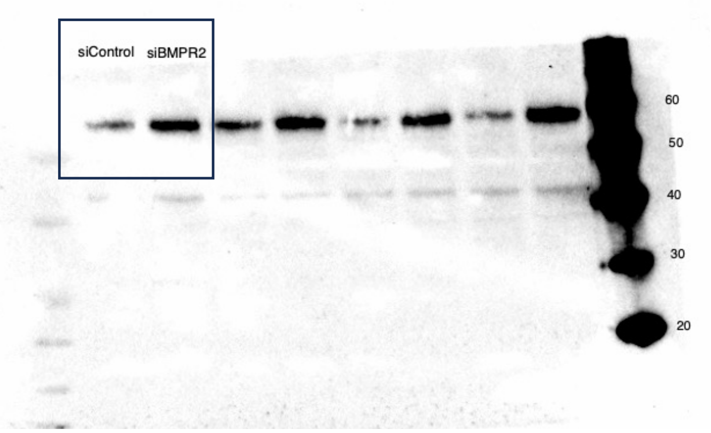

Total AKT

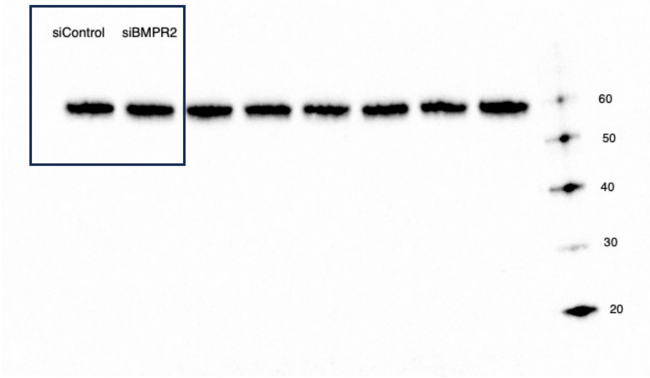

pBAD (S136)

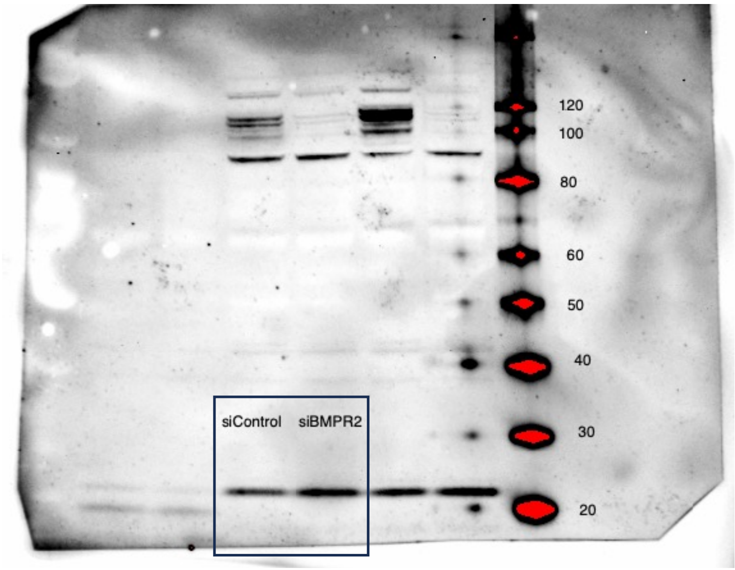

Total BAD

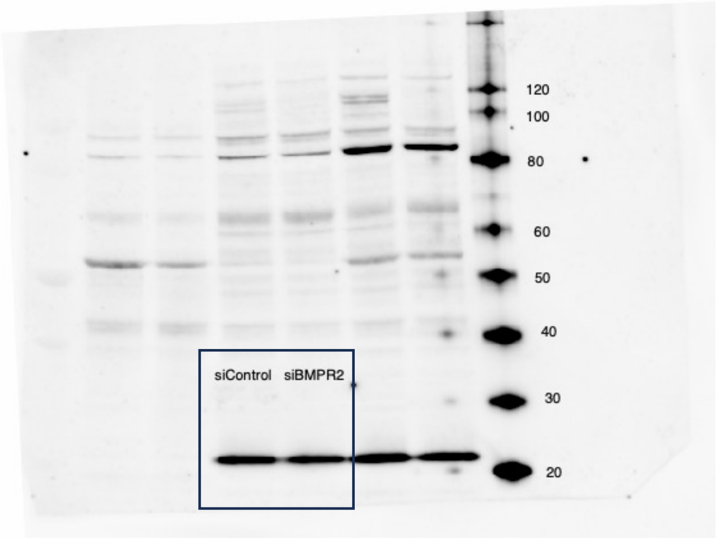

Figure 2A

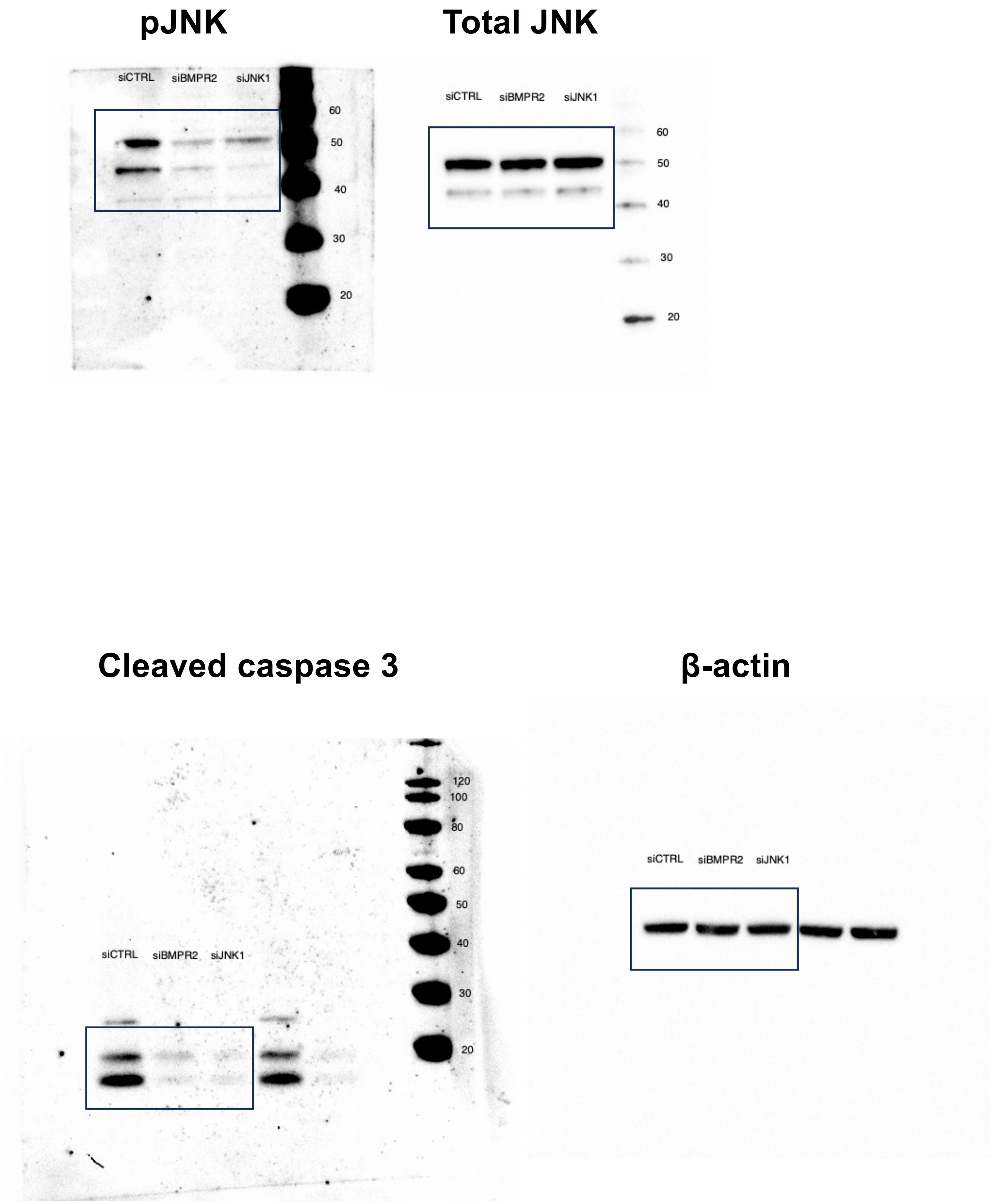
pJNK
siCTRL
siBMPR2
siJNK1
60
50
40
30
20
Total JNK
siCTRL
siBMPR2
siJNK1
60
50
40
30
20
Cleaved caspase 3
120
100
80
60
50
40
30
20
siCTRL
siBMPR2
siJNK1
β-actin
siCTRL
siBMPR2
siJNK1

Figure 2A

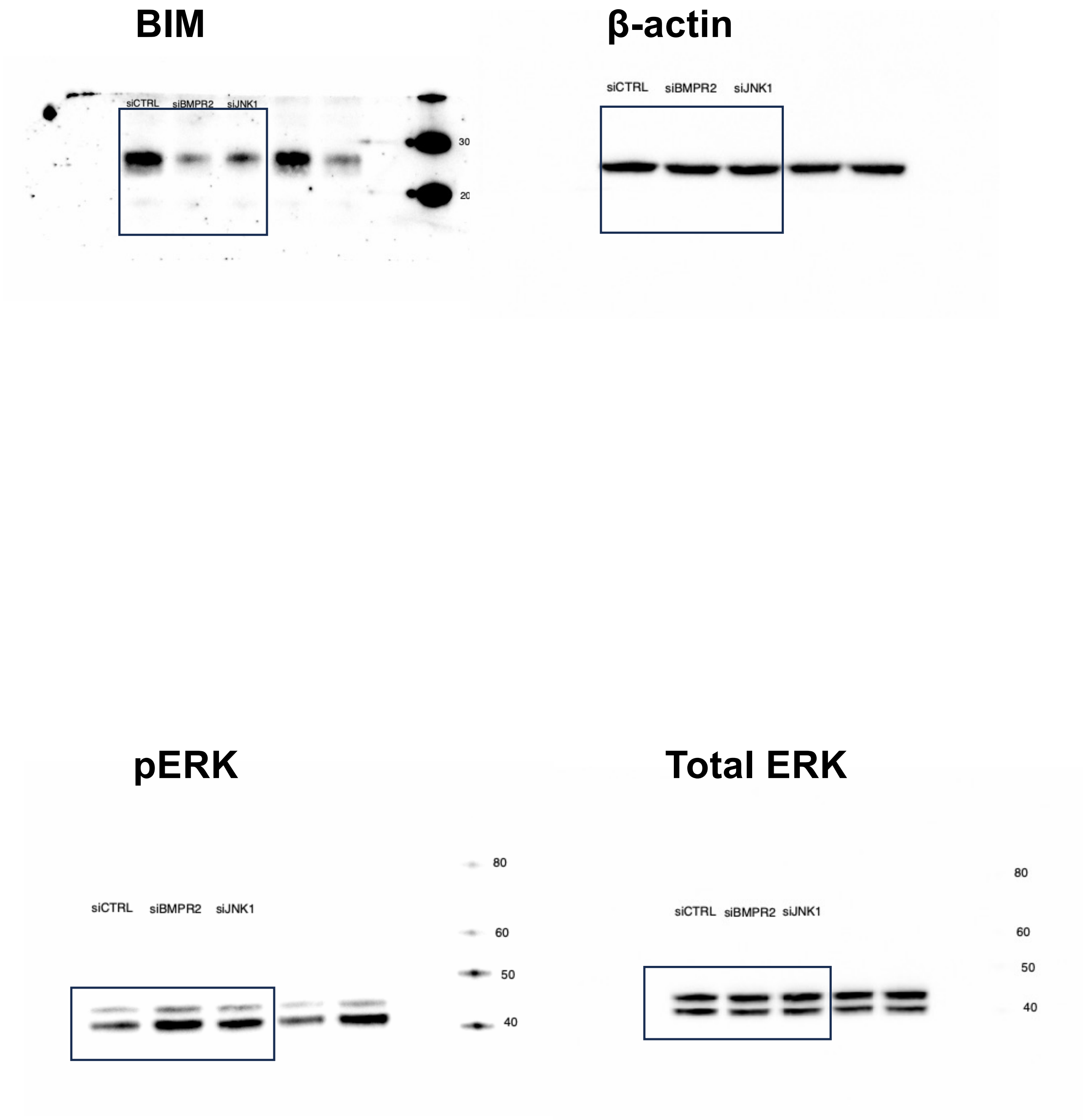
BIM
siCTRL
siBMPR2
siJNK1
30
20
β-actin
siCTRL
siBMPR2
siJNK1
pERK
siCTRL
siBMPR2
siJNK1
80
60
50
40
Total ERK
siCTRL
siBMPR2
siJNK1
80
60
50
40

Figure 3A

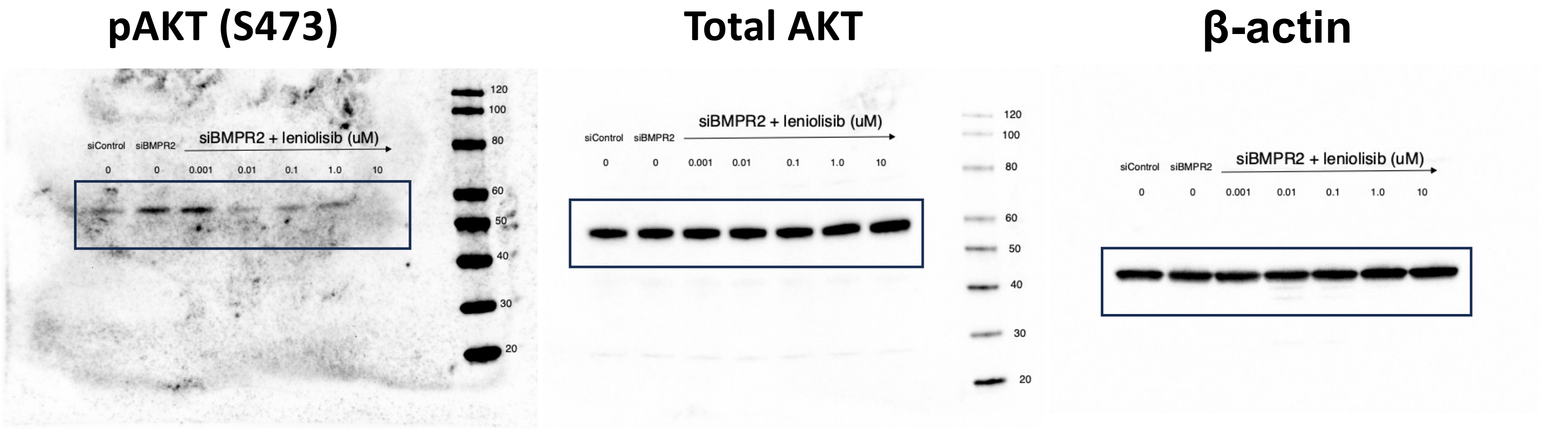
pAKT (S473)
Total AKT
β-actin
siControl
siBMPR2
siBMPR2 + leniolisib (uM)
0
0
0.001
0.01
0.1
1.0
10
120
100
80
60
50
40
30
20

Figure 3B

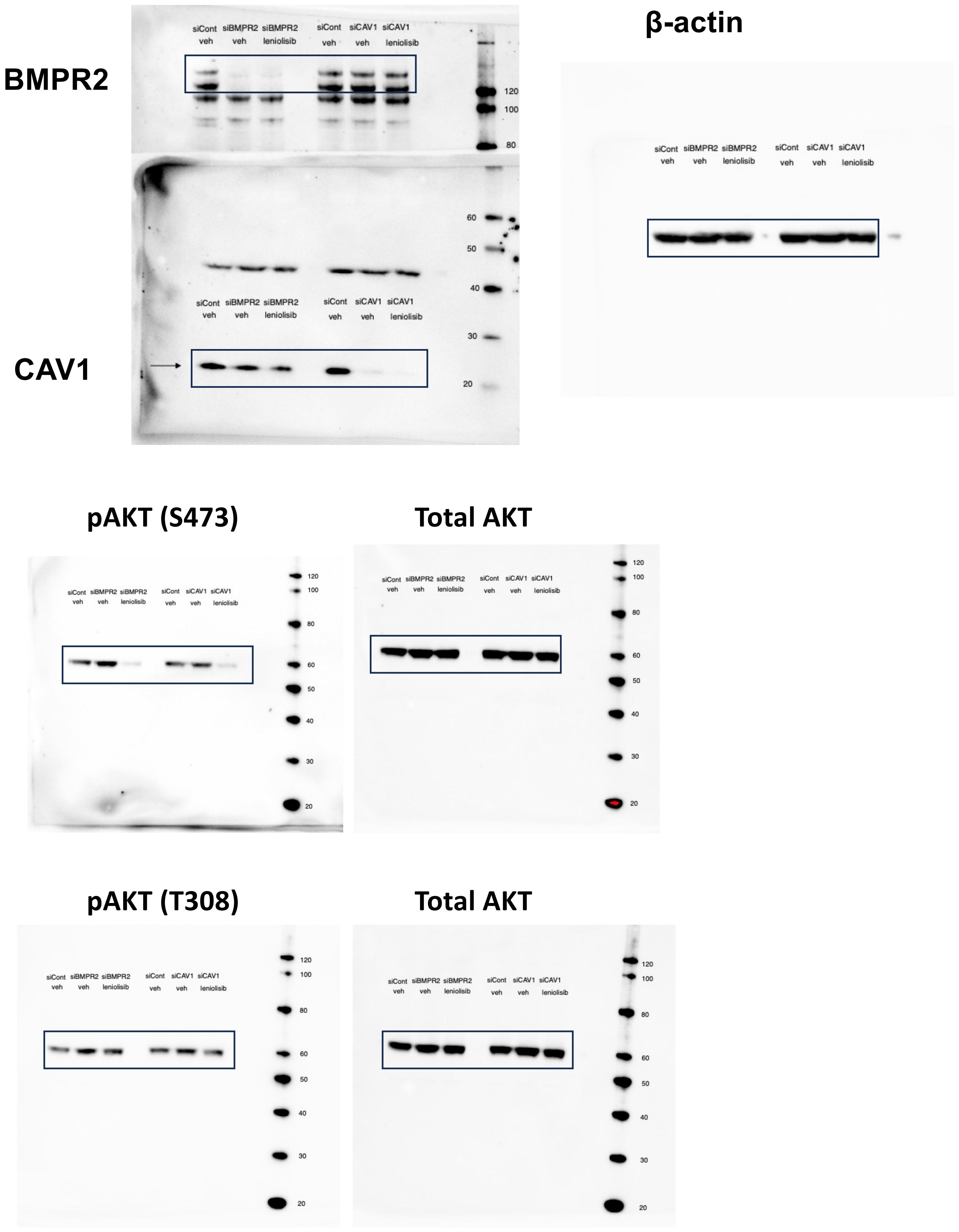
BMPR2
CAV1
β-actin
pAKT (S473)
Total AKT
pAKT (T308)
Total AKT
siCont veh
siBMPR2 veh
siBMPR2 leniolisib
siCont veh
siCAV1 veh
siCAV1 leniolisib
120
100
80
60
50
40
30
20

Figure 4A

DLL4

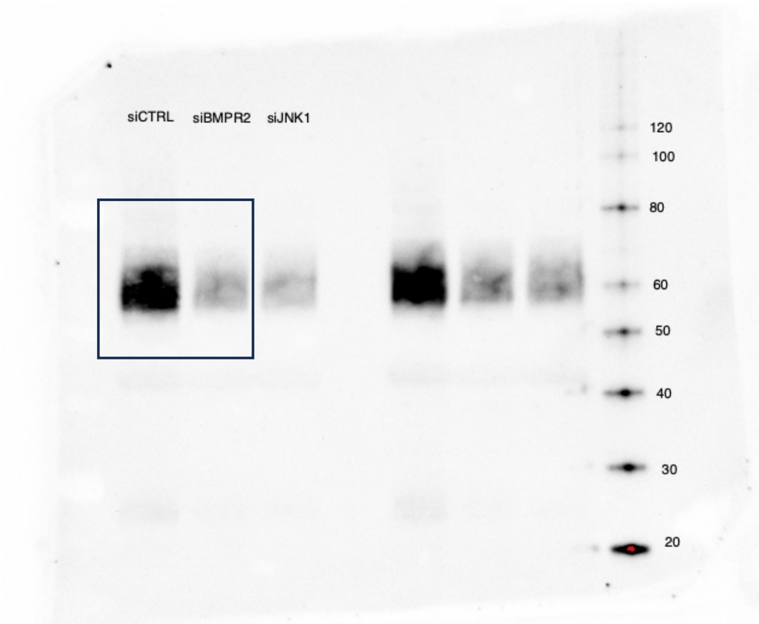

β-actin

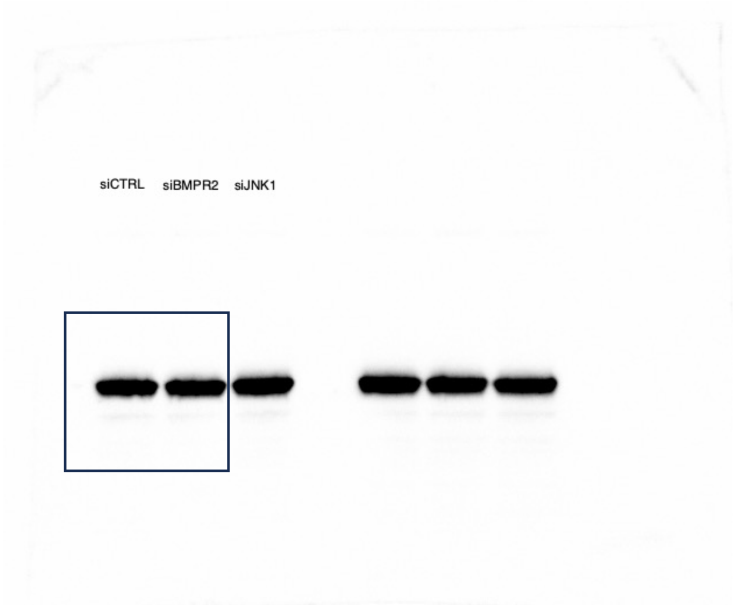

Figure 4B

N1ICD

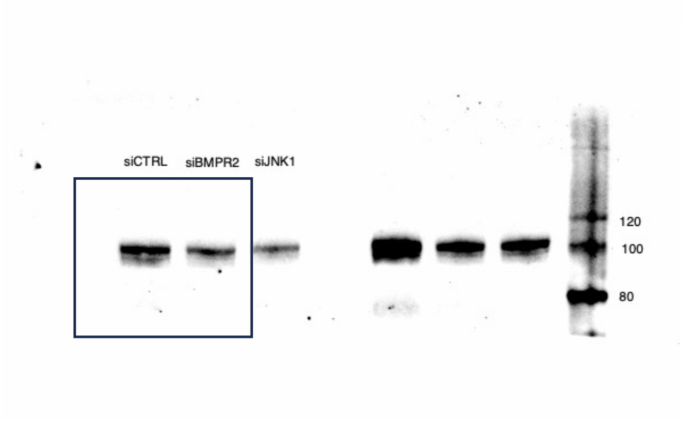

β-actin

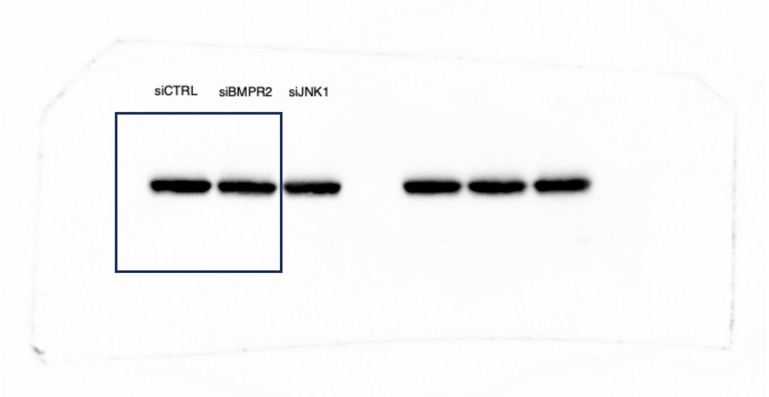

Figure 4C

NOTCH1

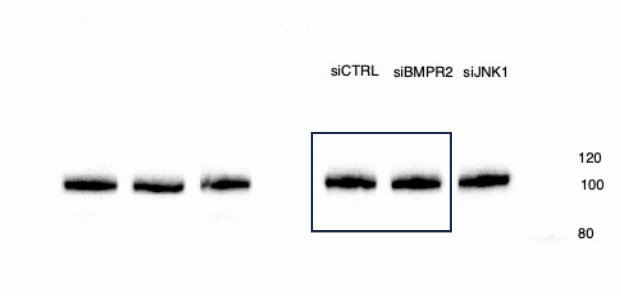

β-actin

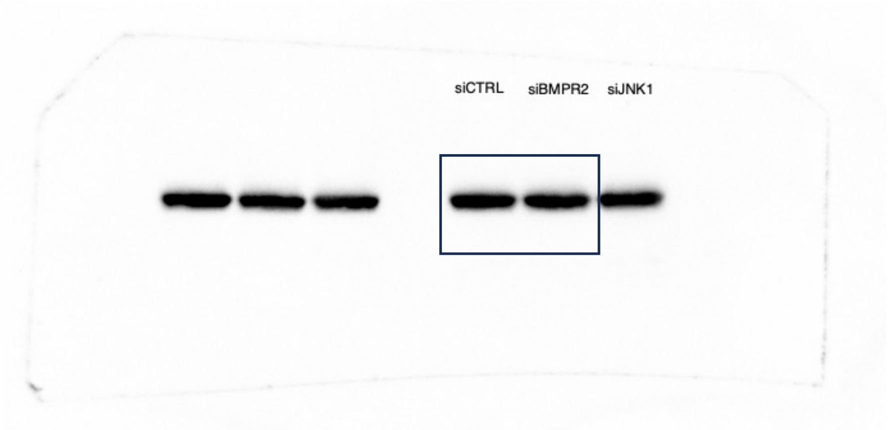

Figure 4D

N2ICD

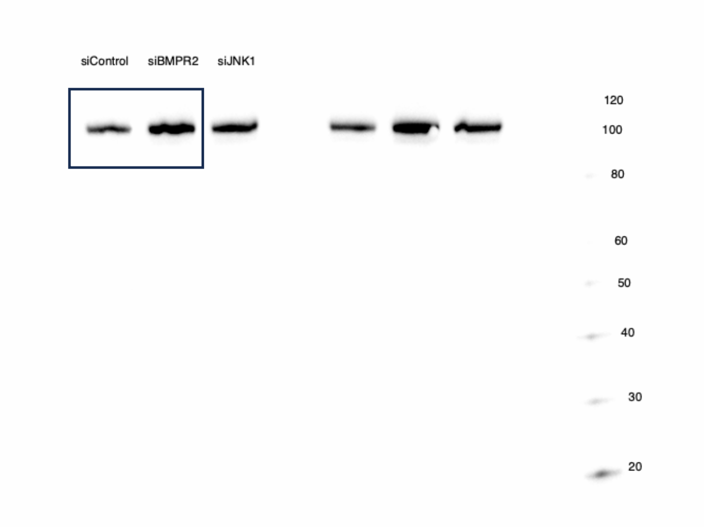

β-actin

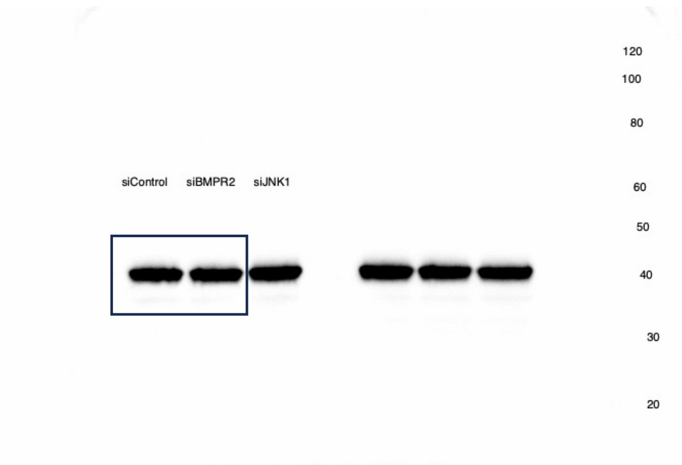

Figure 4E

N4ICD

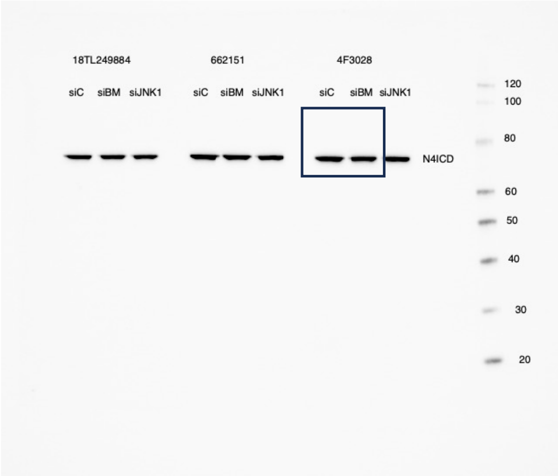

β-actin

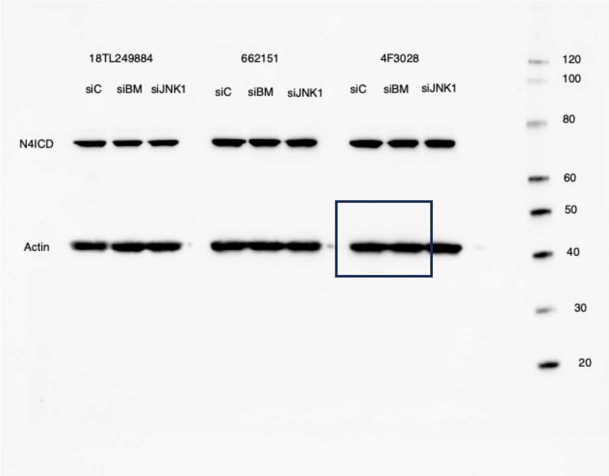

Figure 4F

DLL4

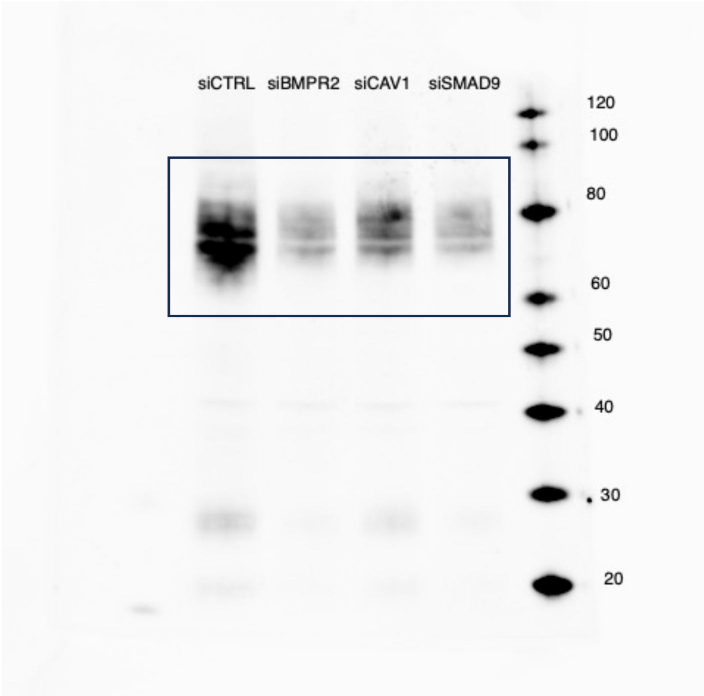

β-actin

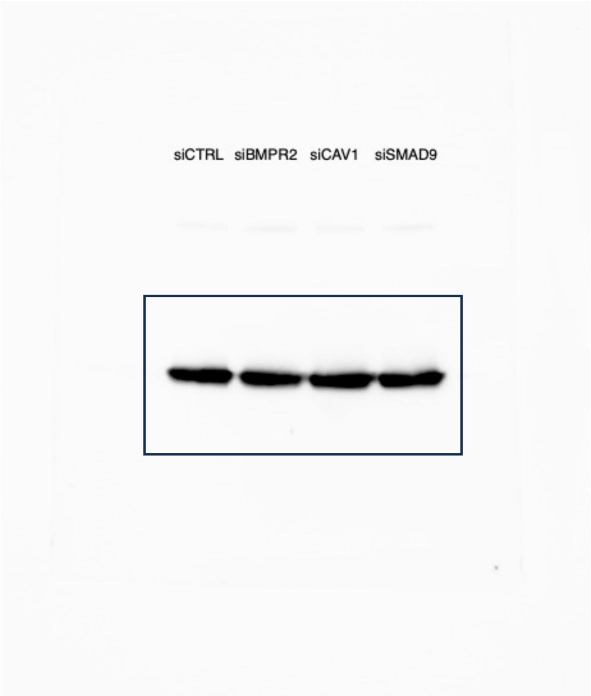

Figure 5A

DLL4

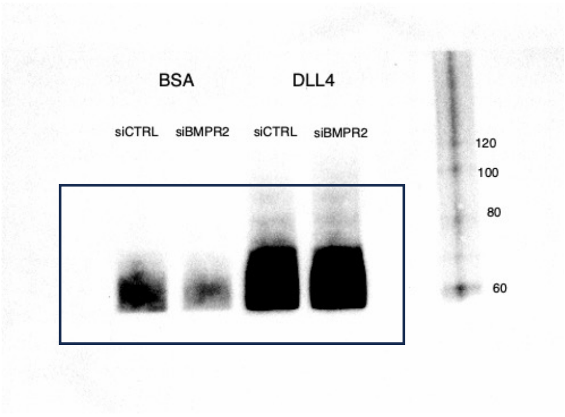

N4ICD

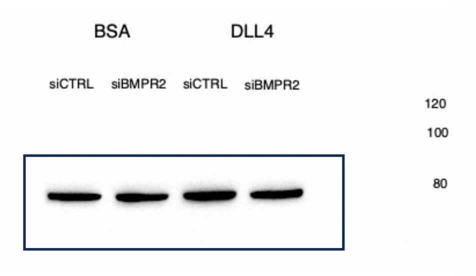

β-actin

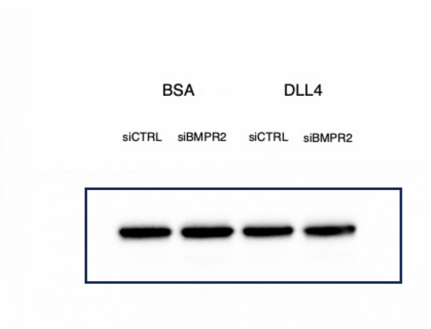

N1ICD

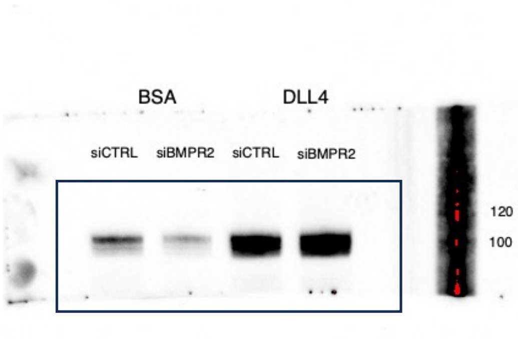

NOTCH1

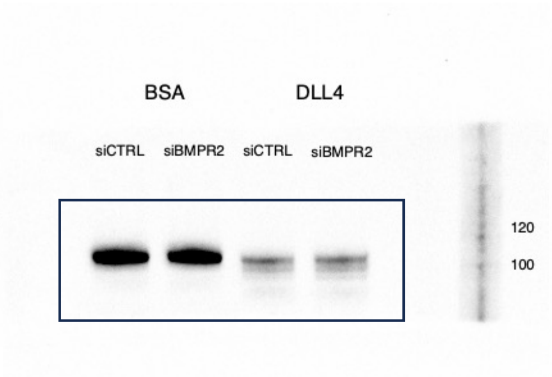

β-actin

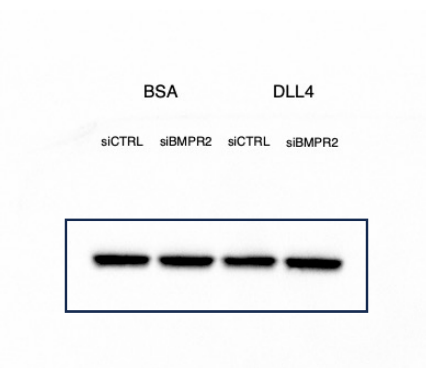

N2ICD

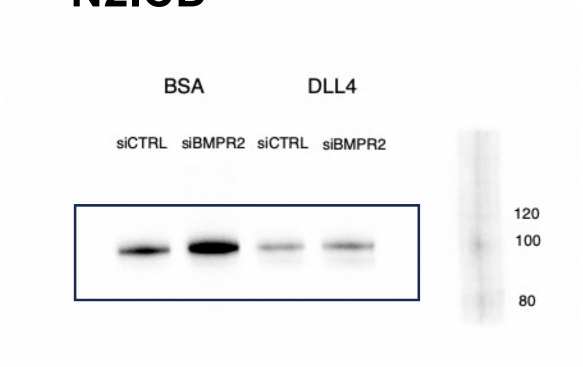

β-actin

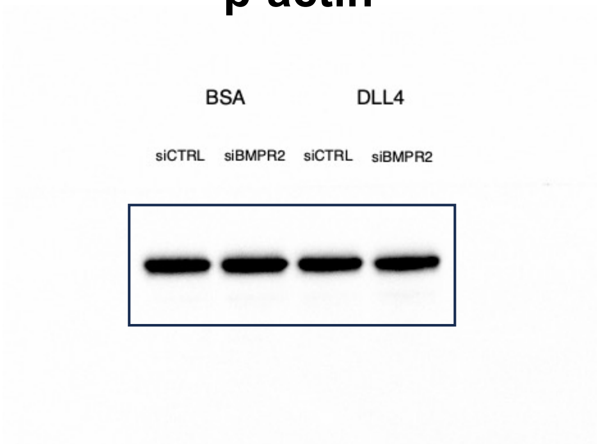

Figure 5B

pAKT (S473)

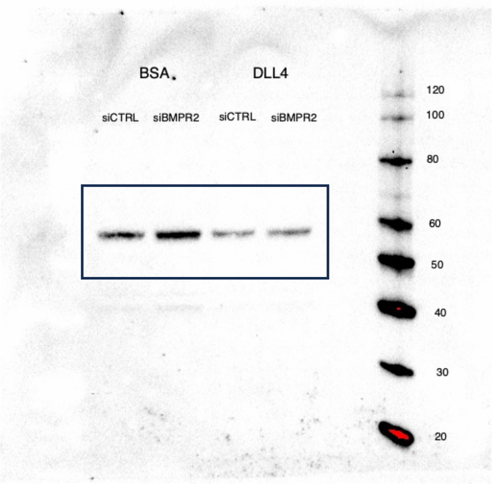

Total AKT

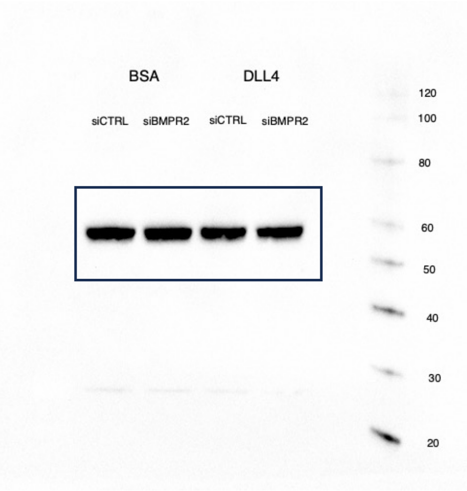

pAKT (T308)

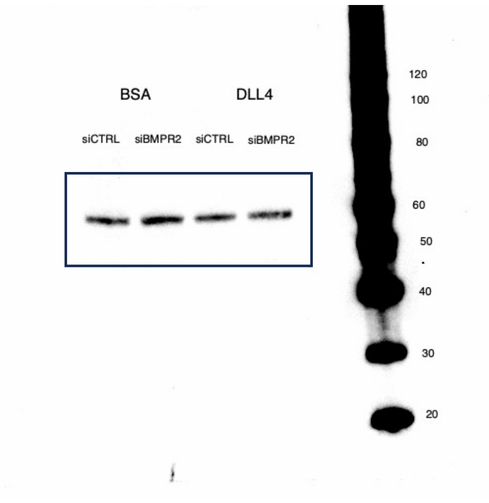

Total AKT

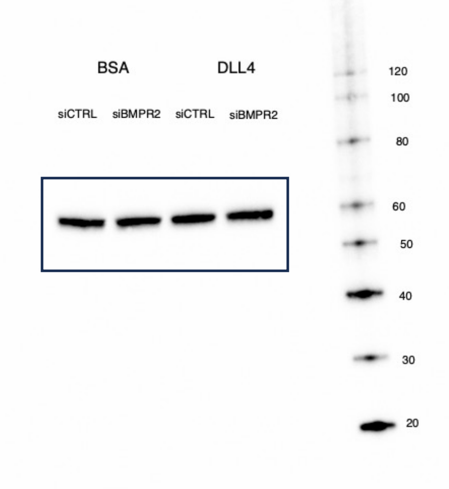

pERK

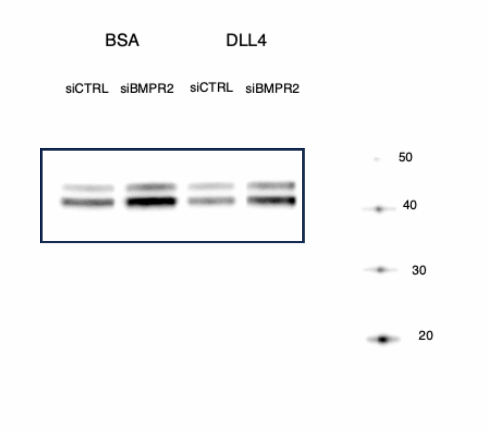

Total ERK

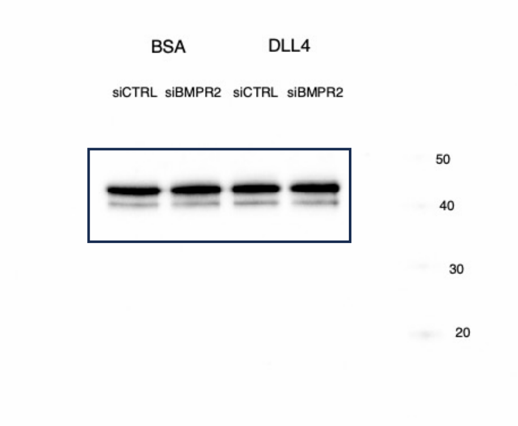

Figure 7E

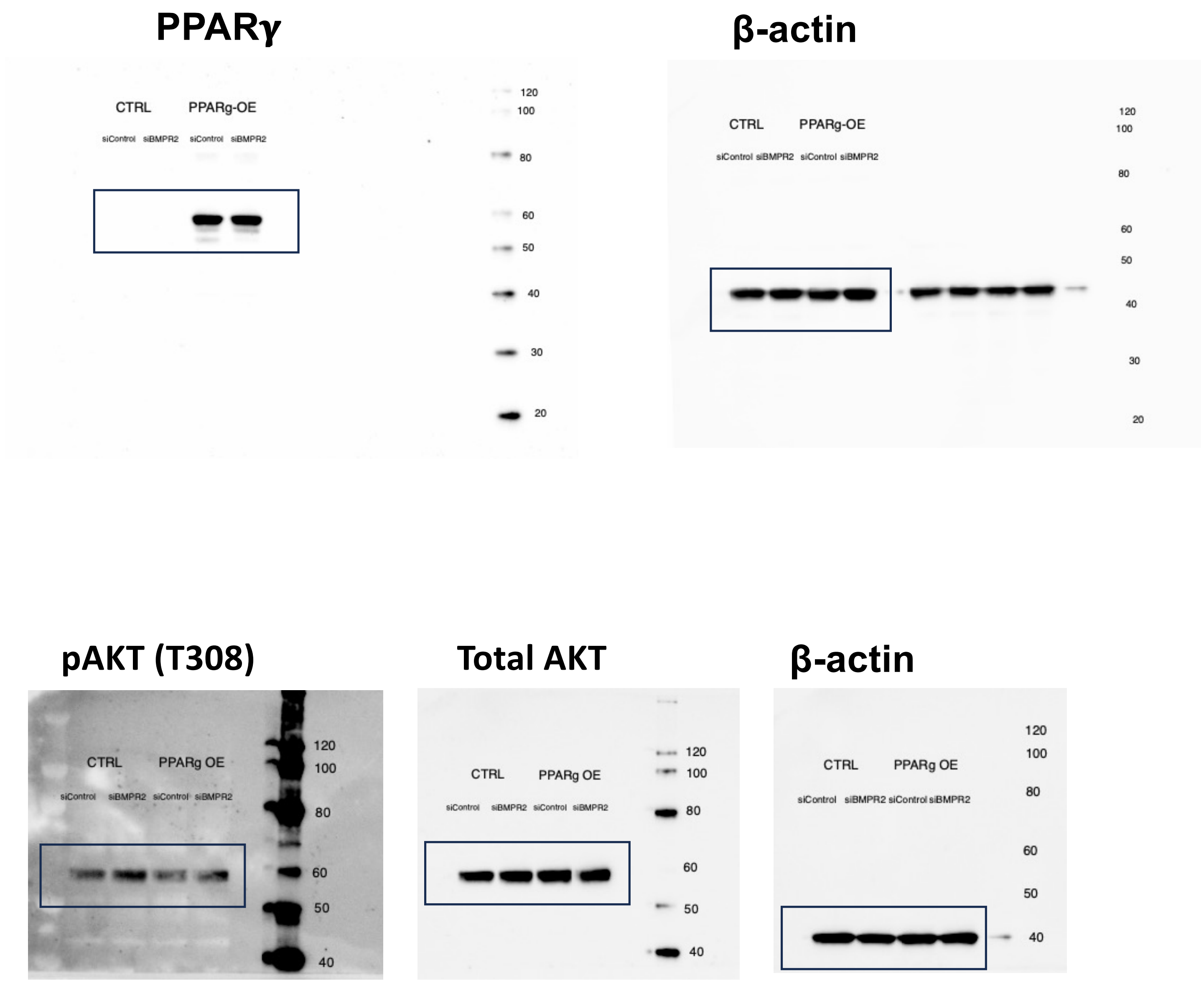

PPARγ
CTRL
PPARg-OE
siControl
siBMPR2
siControl
siBMPR2
120
100
80
60
50
40
30
20
β-actin
CTRL
PPARg-OE
siControl siBMPR2 siControl siBMPR2
120
100
80
60
50
40
30
20
pAKT (T308)
CTRL
PPARg OE
siControl
siBMPR2
siControl
siBMPR2
120
100
80
60
50
40
Total AKT
CTRL
PPARg OE
siControl
siBMPR2
siControl
siBMPR2
120
100
80
60
50
40
β-actin
CTRL
PPARg OE
siControl siBMPR2 siControl siBMPR2
120
100
80
60
50
40

Supplementary Figure 4B

PAECS

α-SMA

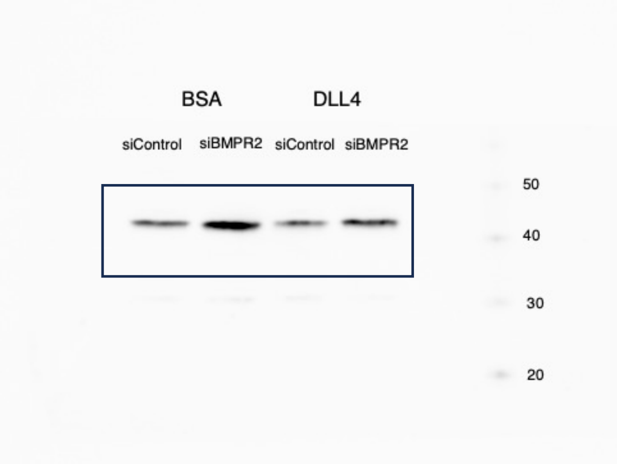

β-actin

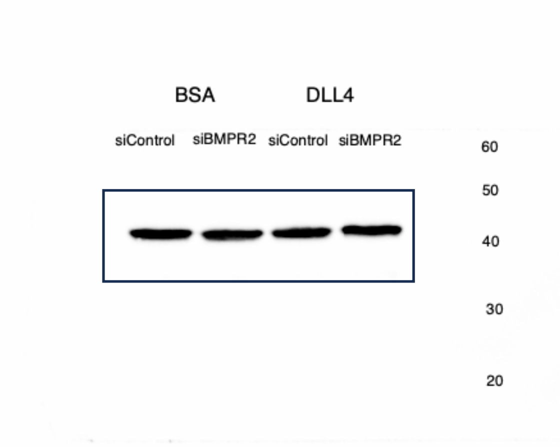

Supplementary Figure 4C

CTRL (FD) AND IPAH

α-SMA

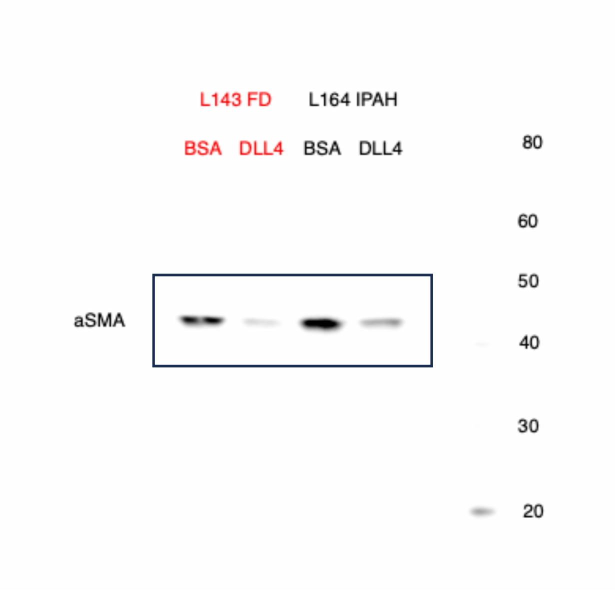

β-actin

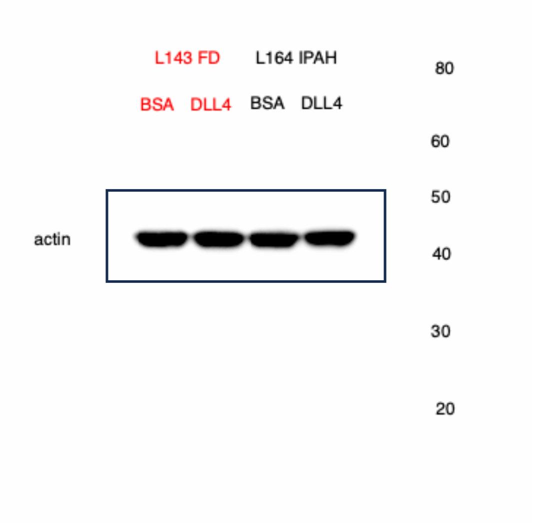

Supplementary Figure 5

DLL4

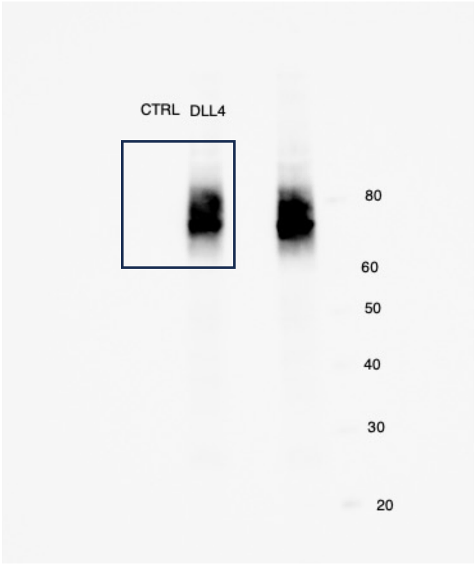

β-actin

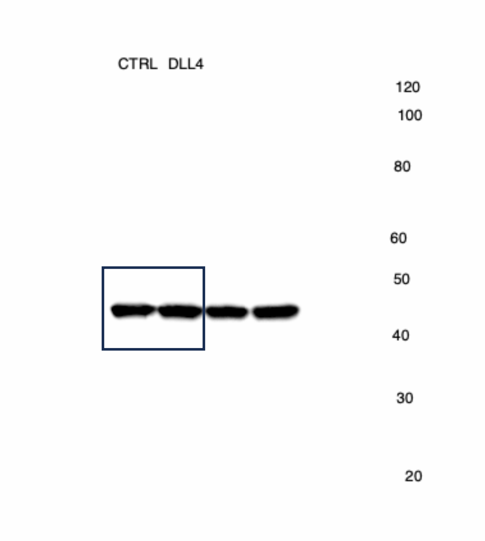

N1ICD

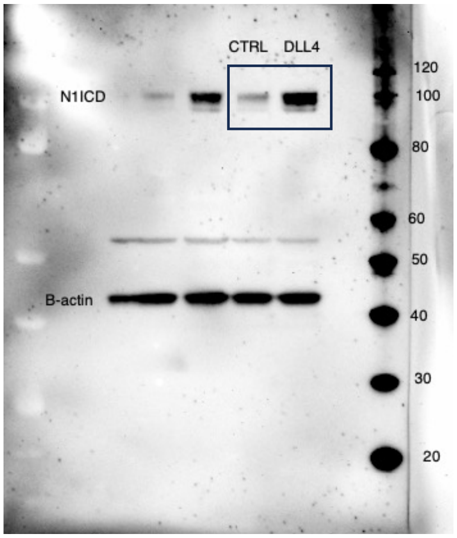

NOTCH1

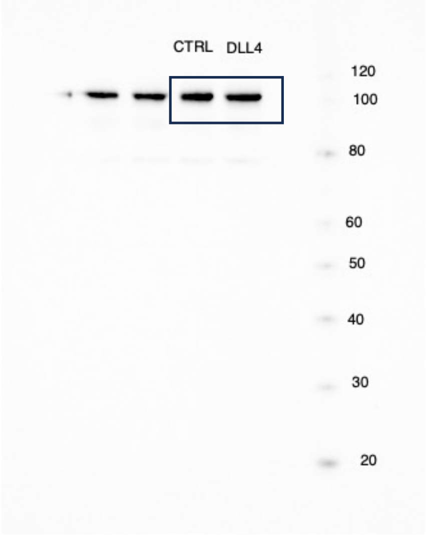

β-actin

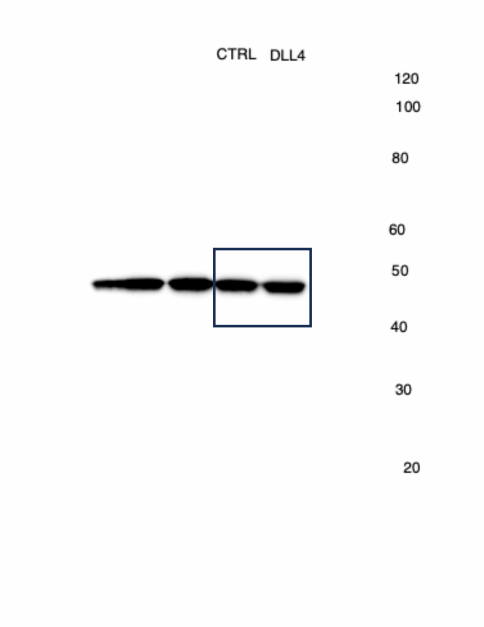

Supplement: Supplementary file 1 [file ijms-25-05403-s001.zip › uncut western blots .pdf]
